# Interplay of gut microbiota and host epithelial mitochondrial dysfunction is necessary for the development of spontaneous intestinal inflammation in mice

**DOI:** 10.1186/s40168-023-01686-9

**Published:** 2023-11-17

**Authors:** Kibrom M. Alula, Alexander S. Dowdell, Brittany LeBere, J. Scott Lee, Cassandra L. Levens, Kristine A. Kuhn, Benny A. Kaipparettu, Winston E. Thompson, Richard S. Blumberg, Sean P. Colgan, Arianne L. Theiss

**Affiliations:** 1 Division of Gastroenterology & Hepatology, University of Colorado Anschutz Medical Campus, 12700 East 19Th Avenue, RC2 Campus Box BB158 HSC, Aurora, CO 80045 USA; 2 Division of Rheumatology, University of Colorado Anschutz Medical Campus, Aurora, CO 80045 USA; 3 Department of Molecular and Human Genetics, Baylor College of Medicine, Houston, TX 77030 USA; 4 Department of Obstetrics and Gynecology, Morehouse School of Medicine, Atlanta, GA USA; 5 Division of Gastroenterology, Department of Medicine, Brigham and Women’s Hospital, Harvard Medical School, Boston, MA USA

**Keywords:** Inflammatory bowel disease, Crohn’s disease, Short-chain fatty acids, Butyrate, Enteroids, Mitochondria, Antimicrobial peptides

## Abstract

**Background:**

Intestinal epithelial cell (IEC) mitochondrial dysfunction involvement in inflammatory bowel diseases (IBD), including Crohn’s disease affecting the small intestine, is emerging in recent studies. As the interface between the self and the gut microbiota, IECs serve as hubs of bidirectional cross-talk between host and luminal microbiota. However, the role of mitochondrial-microbiota interaction in the ileum is largely unexplored. Prohibitin 1 (PHB1), a chaperone protein of the inner mitochondrial membrane required for optimal electron transport chain function, is decreased during IBD. We previously demonstrated that mice deficient in PHB1 specifically in IECs (*Phb1*^*i∆IEC*^) exhibited mitochondrial impairment, Paneth cell defects, gut microbiota dysbiosis, and spontaneous inflammation in the ileum (ileitis). Mice deficient in PHB1 in Paneth cells (epithelial secretory cells of the small intestine; *Phb1*^*∆PC*^) also exhibited mitochondrial impairment, Paneth cell defects, and spontaneous ileitis. Here, we determined whether this phenotype is driven by *Phb1* deficiency-associated ileal microbiota alterations or direct effects of loss of PHB1 in host IECs.

**Results:**

Depletion of gut microbiota by broad-spectrum antibiotic treatment in *Phb1*^*∆PC*^ or *Phb1*^*i∆IEC*^ mice revealed a necessary role of microbiota to cause ileitis. Using germ-free mice colonized with ileal microbiota from *Phb1*-deficient mice, we show that this microbiota could not independently induce ileitis without host mitochondrial dysfunction. The luminal microbiota phenotype of *Phb1*^*i∆IEC*^ mice included a loss of the short-chain fatty acid butyrate. Supplementation of butyrate in *Phb1-*deficient mice ameliorated Paneth cell abnormalities and ileitis. *Phb1*-deficient ileal enteroid models suggest deleterious epithelial-intrinsic responses to ileal microbiota that were protected by butyrate.

**Conclusions:**

These results suggest a mutual and essential reinforcing interplay of gut microbiota and host IEC, including Paneth cell, mitochondrial health in influencing ileitis. Restoration of butyrate is a potential therapeutic option in Crohn’s disease patients harboring epithelial cell mitochondrial dysfunction.

Video Abstract

**Supplementary Information:**

The online version contains supplementary material available at 10.1186/s40168-023-01686-9.

## Introduction

Crohn’s disease is a type of inflammatory bowel disease (IBD) that is marked by chronic inflammation of the gastrointestinal (GI) tract, abdominal pain, diarrhea, and fatigue [1]. Crohn’s disease can affect the entire GI tract with discontinuous, transmural inflammation including characteristic histological granulomas, with ~ 75% of Crohn’s disease patients exhibiting inflammation involving the distal small intestine (the ileum). The prevalence of Crohn’s disease has been increasing steadily worldwide with the highest increase in the USA and other Westernized nations [2]. Although the etiology of IBD is not known, it is associated with complex genetic susceptibility, immune hyperactivity in response to gut microbiota, and environmental factors that can vary by geographical location.

It is well-accepted that IBD is associated with decreased diversity in the intestinal microbiota [3, 4]. Host intestinal epithelial cells (IECs) such as goblet cells and Paneth cells secrete factors such as mucins and antimicrobial peptides (AMPs) that contribute to the maintenance of a healthy relationship between gut microbiota and the intestinal tissue. Paneth cells are a crucial source of AMPs, including lysozyme, α-defensins (cryptdins in mice), and secretory phospholipase A_2_, that shape the gut microbiota composition [5]. Several Crohn’s disease susceptibility genes converge on Paneth cell dysfunction including *ATG16L1*, *LRRK2*, *IRGM*, *XBP1*, and *NOD2*, and there is a lack of Paneth cell α-defensins in ileal Crohn’s disease [6, 7]. Microbe-derived metabolites include short-chain fatty acids (SCFAs), of which the most abundant are acetic, propanoic, and butyric acids that are produced via bacterial fermentation and interact with IECs as well as the mucosal immune cells to promote intestinal homeostasis [8, 9]. Butyrate is the most studied SCFA and has been shown to play a role in maintaining barrier integrity, dampening inflammation by modulating the immune system, altering histone deacetylase (HDAC) activity, and providing a carbon energy source to IECs [10]. Loss of butyrate synthesis has been noted in IBD patients [11].

Emerging studies implicate the involvement of mitochondrial dysfunction in IBD [12–16]. Consistent with the mitochondria serving as the powerhouse of the cell, it has long been proposed that failure to procure energy from the microbiota (i.e., “the starved gut hypothesis”) is a driving force in the pathogenesis of IBD [17, 18]. We recently demonstrated that during active Crohn’s disease inflammation, the epithelium exhibited mitochondrial damage evident in Paneth cells, goblet cells, and enterocytes [19]. We previously generated a mouse model of intestinal epithelial mitochondrial dysfunction via the deletion of PHB1 (*Phb1*^*i∆IEC*^ mice) [20], which is a chaperone protein required for optimal activity of the electron transport chain that exhibits a loss of expression in mucosal biopsies of IBD-afflicted patients [21–23]. *Phb1*^*i∆IEC*^ mice developed spontaneous ileitis that was preceded by mitochondrial dysfunction in all epithelial cells, gut microbiota dysbiosis, and early abnormalities in Paneth cells [20]. Abnormalities in Paneth cells of *Phb1*^*i∆IEC*^ mice included decreased expression of AMPs, altered lysozyme staining patterns, and increased alcian blue and muc2 staining, suggesting that Paneth cells in these mice take on an “intermediate” goblet/Paneth cell type [20]. Deletion of PHB1 specifically in Paneth cells driven by *Defα6*^*Cre*^ or *Mist1*^*CreERT2*^ (*Phb1*^*∆PC*^) was sufficient to induce Paneth cell defects and ileitis in mice. Twenty to 50% of Crohn’s disease patients manifest Paneth cell defects that correlate with worse clinical outcomes and gut microbiota dysbiosis [24–26]. Independent of inflammation, Crohn’s disease patients with Paneth cell defects exhibit a high proportion of altered mitochondria in these cells [19]. Nevertheless, the role of gut microbiota in driving inflammation during impaired IEC or Paneth cell mitochondrial health is not well-elucidated. In the present study, we investigated the role of the mitochondrion-microbiome axis in mouse models of spontaneous ileitis driven by the loss of *Phb1* expression.

## Materials and methods

### Animal models

*Phb1*^*fl/fl*^, *Phb1*^*ΔPC*^, and *Phb1*^*iΔIEC*^ C57BL/6 J mice with constitutive PHB1 deletion in Paneth cells (*Phb1*^*ΔPC*^ driven by Defα6-Cre) or inducible Phb1 deletion in intestinal epithelial cells (*Phb1*^*iΔIEC*^ driven by Villin-CreER^T2^) were previously described [20]. All experiments were conducted with age- and gender-matched littermate mice under co-housing or alone-housing by genotype conditions based on the individual experiment. All experiments were approved by the University of Colorado Anschutz Medical Campus Institutional Animal Care and Use Committee.

### Induction of Phb1 deletion from intestinal epithelial cells

Eight-week-old *Phb1*^*fl/fl*^* and Phb1*^*iΔIEC*^ male and female littermate mice were intraperitoneally injected daily with 100 µl of 10 mg/ml tamoxifen (Sigma-Aldrich, St. Louis, MO) for 4 consecutive days to induce deletion of the *Phb1* gene. Tamoxifen injections were repeated every 3–4 weeks to guarantee continuity of PHB1 deletion [27]. Body weight was measured weekly. Mice were sacrificed 12 weeks following the initial tamoxifen injection.

### 16S rRNA sequencing

Ileal luminal contents were collected from *Phb1*^*fl/fl*^, *Phb1*^*ΔPC*^, and *Phb1*^*iΔIEC*^ mice at 20 weeks of age upon sacrifice. Bacterial DNA was extracted using the Qiagen DNeasy PowerSoil Pro DNA isolation Kit (Maryland, USA) according to the manufacturer’s instructions with two additions to enhance cell lysis. First, a 10-min incubation at 65 °C was added prior to manual agitation, and second, a benchtop homogenizer was used in place of vortexing. The amplicon library was prepared for the 16S rRNA V4 region, and the library was constructed using Illumina DNA prep. Quality control for the completed libraries was conducted using a combination of PicoGreen (Thermo), Qubit (Invitrogen), and Fragment Analyzer (Agilent, and Tapestation (Agilent). Twenty-five cycles were used for PCR amplification. As a part of the internal QC process and at each step of the sequencing process, positive (20-strain bacterial mock community control sample: MSA2002, ATCC) and negative (blank) controls were included. Data were demultiplexed using bbduk.sh (BBMap, version 38.82), removing Illumina adapters, PhiX reads, and reads with a Phred quality score < 15 and length < 100 bp after trimming. All chimeric reads were screened out, and quality-controlled reads were merged using bbmerge.sh. Then, all reads were combined into a single FASTA file for further analysis. Raw data files in binary base call (BCL) format are converted into FASTQs and demultiplexed based on the single-index barcodes using the Illumina bcl2fastq software (CMMR, Baylor College of Medicine; https://data.jplab.net/EipsKgQV/index.html).

For studies using germ-free rederivation, the V4 region (515f.-806r; FWD: GTGCCAGCMGCCGCGGTAA; REV: GGACTACHVGGGTWTCTAAT) of the 16S rDNA gene was amplified using Accustart II PCR SuperMix (Quantabio). Primer construction and amplification followed the Earth Microbiome Project (www.earthmicrobiome.org) protocol. Amplified barcoded DNA fragments were quantified using a PicoGreen assay (Invitrogen) and equal amounts (ng) of DNA from each sample were pooled. The aggregate pool was sequenced using a V2 2 × 250 kit on the Illumina MiSeq platform (San Diego, CA) at the University of Colorado Anschutz Medical Campus using the Mucosal Immunobiology Core, Center for Mucosal Immunology and Rheumatic Disease Pathogenesis (CMIR). 16S rRNA amplicons were demultiplexed using QIIME2 and then denoised into ASVs using DADA2.

### Gas chromatography-mass spectrometry (GC–MS) of short-chain fatty acids (SCFAs)

Using the Analytical Resource Core (ARC) – Bioanalysis and Omics, Colorado State University, short-chain fatty acids were determined by GC–MS using the method described by Lotti et al. [28] with modifications. Briefly, fecal water was prepared on dry ice by weighing ileal content samples into a 2-ml homogenization tube with a stainless steel bead. An equal volume of ice-cold PBS was added, and the sample was homogenized on a Qiagen Tissuelyser (Qiagen, Germantown, MD) for 2 min at 50 Hz. The samples were centrifuged at 14,000 × g for 10 min at 4 °C, and the supernatant was removed and either prepared for GC/MS analysis immediately or frozen at − 70 °C.

The fecal water was prepared for GC/MS analysis by aliquoting 50 µl into a 2-ml microcentrifuge tube, adding 10 µl of acidified water (15% phosphoric acid), 20 µl of internal standard (45-µM acetic acid-d4, 10-mM propanoic acid-d6, and 10-mM butyric acid-d7 in methyl tert butyl ether), and 1 ml of methyl tert butyl ether (MTBE). The samples were vortexed vigorously for ~ 5 s and then placed on an orbital shaker for 5 min. The aqueous and organic phases were allowed to separate, and then, the upper organic (MTBE) layer was transferred to an amber autosampler vial with a screw cap. GC–MS analysis was performed on an Agilent 7890 GC interfaced with a 5977 MS (Agilent Technologies, Santa Clara, CA) operated in EI mode. Separation of SCFAs was performed on an Agilent DB-FATWAX 30 m × 0.25 mm column with helium as carrier gas. Data processing and quantitative analysis were performed using Agilent MassHunter Quantitative analysis software.

### Conventionalized germ-free mice

In collaboration with CU Anschutz Gnotobiotic Core, 8-week-old germ-free (GF) mice were conventionalized with specific pathogen-free (SPF) gut microbiota from alone-housed 20-week-old *Phb1*^*fl/fl*^ or *Phb1*^*i∆IEC*^ mice. Donor SPF microbiota consisted of 100 mg of ileal contents from 4 *Phb1*^*fl/fl*^ or *Phb1*^*i∆IEC*^ mice pooled that were homogenized in an anaerobic chamber in 1 ml reduced 1 × PBS (has been stored in an anaerobic chamber to remove oxygen). After sedimentation by gravity, 150 µL of the slurry was orally gavaged into recipient GF mice gender-matched to the donor gender. Following gavage, recipient mice were maintained in gnotobiotic conditions, weighed once weekly for 12 weeks, and ileal luminal contents and tissue were then collected for 16S rRNA sequencing and measurements of inflammation, respectively. To ensure donor ileal contents conventionalized GF mice, 16S rRNA sequencing was performed on the ileal contents of GF recipient mice and compared to 16S rRNA sequencing of donor contents obtained at the time of gavage.

### Antibiotics treatment

A broad-spectrum antibiotic cocktail was used to deplete gut microbiota from SPF *Phb1*^*∆PC*^, *Phb1*^*i∆IEC*^, and *Phb1*^*fl/fl*^ littermates. Mice were treated with 1 g/L ampicillin sodium salt, 1 g/L neomycin sulfate, 1 g/L metronidazole, and 500 mg/L vancomycin in autoclaved drinking water for 4 weeks beginning at 16 weeks of age and compared to untreated mice.

### Butyrate supplementation

Mice were administered a 20-mM sodium butyrate (Sigma-Aldrich, 303,410) in the drinking water from 16–20 weeks of age. This dose was chosen based on previous studies showing intestinal epithelial response [29].

### 3D ileal enteroid culture and treatment with ileal microbiota filtrates

Enteroids were harvested and cultured from 8-week-old ileal crypts of *Phb1*^*fl/fl*^, *Phb1*^*ΔPC*^, and *Phb1*^*iΔIEC*^ mice in Matrigel (BD Biosciences, 356,230) as previously described [20]. *Phb1*^*iΔIEC*^ mice were intraperitoneally injected daily with 100 µl of 10 mg/ml tamoxifen for 4 consecutive days prior to harvesting the ileum for enteroid culturing. Enteroids were established from a 7-in. length of the ileum and were grown in complete crypt culture ENR media containing growth factors EGF, Noggin, and R-spondin (obtained from the Organoid and Tissue Modeling Shared Resource core, University of Colorado Anschutz Medical Campus) for 7 days to allow epithelial cell differentiation [30]. On day 7 of culture, enteroids were treated with 200 mg/ml ileal microbiota filtrates for 16 h as previously described for cecal microbiota filtrates [31] and compared to enteroids untreated with ileal microbiota filtrates (vehicle control group). The vehicle consisted of 1 × PBS. Ileal luminal contents were collected from 20-week-old *Phb1*^*fl/fl*^, *Phb1*^*ΔPC*^, or *Phb1*^*iΔIEC*^ mice. Ileal luminal contents were dissolved in 1 × PBS and filtered using a 0.22-µm filter [31]. Protein concentration was determined by Bradford assay. To determine whether SCFAs can ameliorate epithelial responses to microbiota, a subset of enteroids were supplemented with 0.5 mM propionate, 0.5 mM acetate, 0.5 mM butyrate, alone or in combination, concurrent with microbiota filtrate treatment. A subset of enteroids were also treated with 1-µm Mithramycin A (TOCRIS, 1489). Enteroid viability by morphological change was evaluated and quantitated using a ZEISS AXIOSKOPE Plus Inverted Microscope as described previously [32].

### Oxygen consumption rate and reactive oxygen species (ROS) quantification by MitoSOX

Ileal crypts from *Phb1*^*fl/fl*^, *Phb1*^*ΔPC*^, and *Phb1*^*iΔIEC*^ mice were harvested and plated in matrigel on a 24-well OxoDish plate with pre-calibrated oxygen sensors at the bottom of each well (PreSens Precision Sensing), as described previously [33]. As a positive control for altered oxygen consumption, enteroids were treated with 0.1 µM Antimycin A (Sigma-Aldrich, A8674). Concurrent with treatment, the extracellular oxygen consumption rate was measured every minute using the PreSens SDR Sensor Dish Reader. Within 15 min, the oxygen consumption rate plateaued and this time point was graphed to compare treatment groups. Then, enteroids were dispersed into single cells by pipetting up and down in cold trypsin followed by incubation at 37℃ for 3 min. Cells were then pelleted by centrifuging at 200 × g for 5 min after equal volume of DMEM media. Pellets were resuspended in DMEM and plated in 96-well plates. One hundred microliters of 5-µM MitoSOX reagent (Invitrogen, M36008) was added directly to the enteroid culture media after enteroids were dissociated into single cells and incubated for 10 min. MitoSOX Red mitochondrial superoxide fluorescence was read at excitation/emission of 510/580 nm by a plate reader (BioTek, SYNERGY H1 microplate reader). Cell number was quantitated using Cellometer Auto T4 (Nexcelom Bioscience), and relative oxygen consumption and MitoSOX fluorescence was normalized to cell number per well.

### Total histological inflammation scoring

Six micrometers of paraffin-embedded Swiss-rolled ileal sections was stained with hematoxylin and eosin. Histological inflammation scoring was performed in a blind fashion using previously published criteria for mouse ileitis [34], and imaging was performed using ZEISS AXIO microscope and Zen 2.3 Pro software.

### Immunofluorescent staining

Six micrometers of paraffin-embedded Swiss-rolled ileal sections was dehydrated in xylene and ethanol gradient and washed with tap water, and the epitope was retrieved with citric acid buffer (0.1 M citric acid and 0.1 M sodium citrate) in the pressure cooker for 10 min at 125℃. Tissues were washed with 1 × PBS and incubated with 5% normal donkey serum for 1 h at room temperature. Tissues were incubated with primary antibodies targeting lysozyme (1:500, sc-518058, mouse, Santa Cruz) and muc2 (1:500, PA5-21,329, rabbit, Invitrogen) in 5% blocking normal donkey serum overnight at 4℃. Tissues were washed with 1 × PBS followed by incubation for secondary antibodies (1:500, donkey anti-mouse, 715–296-150; and 1:500, donkey anti-rabbit, 711–096-152; Jackson ImmunoResearch) for 1 h at room temperature. Tissues were washed with 1 × PBS and incubated with DAPI (1:1000 in 1 × PBS) for 1 min. Tissues were coverslipped with Prolong Gold Antifade reagent (Invitrogen, P36930). Imaging was performed using ZEISS AXIO microscope and Zen 2.3 Pro software. Lysozyme patterns from Paneth cells were scored normal, disordered, diminished (depleted), or diffuse as previously described [25].

### Alcian blue-periodic acid Schiff staining

Six micrometers of paraffin-embedded ileal sections was stained with Alcian blue (AB; Sigma Aldrich, B8438) solution, pH 2.5, for 5 min and washed in running tap water for 5 min. Sections were then stained with periodic acid and Schiff’s (PAS) reagent according to the PAS kit (395B-1KT, Sigma Aldrich) protocol and mounted using xylene-based mounting media.

### SDS-PAGE and western immunoblot analysis

Total protein was isolated from 5-mm distal ileum, concentration determined by a Bradford assay, separated by SDS-PAGE, and analyzed by western blotting as described previously [20]. The following proteins were probed for by chemiluminescence: Caspase 3 (1:1000, 9662; Cell Signaling) and β-actin (1:10,000, A1978; Millipore Sigma). Immunoblot images were acquired with BioRad ChemiDoc MP Imaging System.

### RNA isolation, cDNA amplification, and quantitative real-time PCR analysis

Total RNA was isolated from 5-mm ileum using Trizol (Invitrogen), and cDNA was synthesized using Flex cDNA Kit (Quantabio, 95,049). Quantitative real-time PCR was performed with QuantStudio 3 (Applied Biosystems), and graphical representation of quantitative real-time PCR data was calculated as follows: ΔΔC_T_ = (C_t_, _target_ – C_t_, _β-actin_)_*Phb1iΔIEC or Phb1ΔPC*_ – (C_t_, _target_ – C_t_, _β-actin_)_*Phb1fl/fl*_, with the final graphical data presented from 2^−ΔΔCT^. The qPCR data were normalized to β-actin. Gene expression analysis using the following primers from Integrated DNA Technologies was performed: β-actin sense: 5′-TATGCCAACACAGTGCTGTCTGG-3′ and β-actin antisense: 5′-TACTCCTGCTTGCTGATCCACAT-3′. Lysozyme sense: 5′-TGGCTGACTGGGTGTGTTTA-3′ and antisense: 5′-CGGTCTCCACGGTTGTAGTT-3′. Cryptdin 3 sense: 5′-CCAGGCTGATCCTATCCAAA-3′ and Cryptdin 3 antisense: 5′-GACACAGCCTGGTCGTCTTC-3′. Cryptdin 5 sense: 5′-GGCTGCAAAAGAAGAGAACG-3′ and Cryptdin 5 antisense: 5′-CAGCTGCAGCAGAATACGAA-3′. Ang4 sense: 5′-GAGCCCATGTCCTTTGTTGT-3′ and Ang4 antisense: 5′-GCTTGGCATCATAGTGCTGA-3′. Tnfa sense: 5′-AGGCTGCCCCGACTACGT-3′ and Tnfa antisense: 5′-ACTTTCTCCTGGTATGAGATAGCAAA-3′. Il1b sense: 5′-TCGCTCAGGGTCACAAGAAA-3′ and Il1b antisense: 5′-CATCAGAGGCAAGGAGGAAAAC-3′. Ifng sense: 5′-CAGCAACAGCAAGGCGAAA-3′ and Ifng antisense: 5′-CTGGACCTGTGGGTTGTTGAC-3′.

### Statistical analysis

Data were analyzed using PRISM Version 9.1.2. GraphPad software. Values are presented as mean ± SD. Outliers were identified by ROUT test (*Q* = 1%). Normality was tested by Shapiro–Wilk normality test and Kolmogorov–Smirnov normality test; all data passed normality. Unpaired two-tailed Student’s *t* test was performed for single comparisons. One-way ANOVA or two-way ANOVA with Bonferroni post hoc test was performed for multiple comparisons. For 16S rRNA data analysis, relationships with continuous variables were tested using R’s base function for linear regression models (“lm”). Differences in community composition use the R function vegan:adonis version 2.5.5 to estimate PERMANOVA *p* values. ATIMA heatmaps cluster by complete linkage and were generated via the R package “pheatmap” version 1.0.12. All *p* values were adjusted for multiple comparisons with Benjamini and Hochberg’s formula to control for the false discovery rate. ATIMA is available at https://atima.research.bcm.edu. *P* < 0.05 was considered statistically significant.

## Results

### Depletion of gut microbiota by broad-spectrum antibiotics ameliorates ileal inflammation and restores Paneth cell health in Phb1-deficient mice

**Fig. 1:**
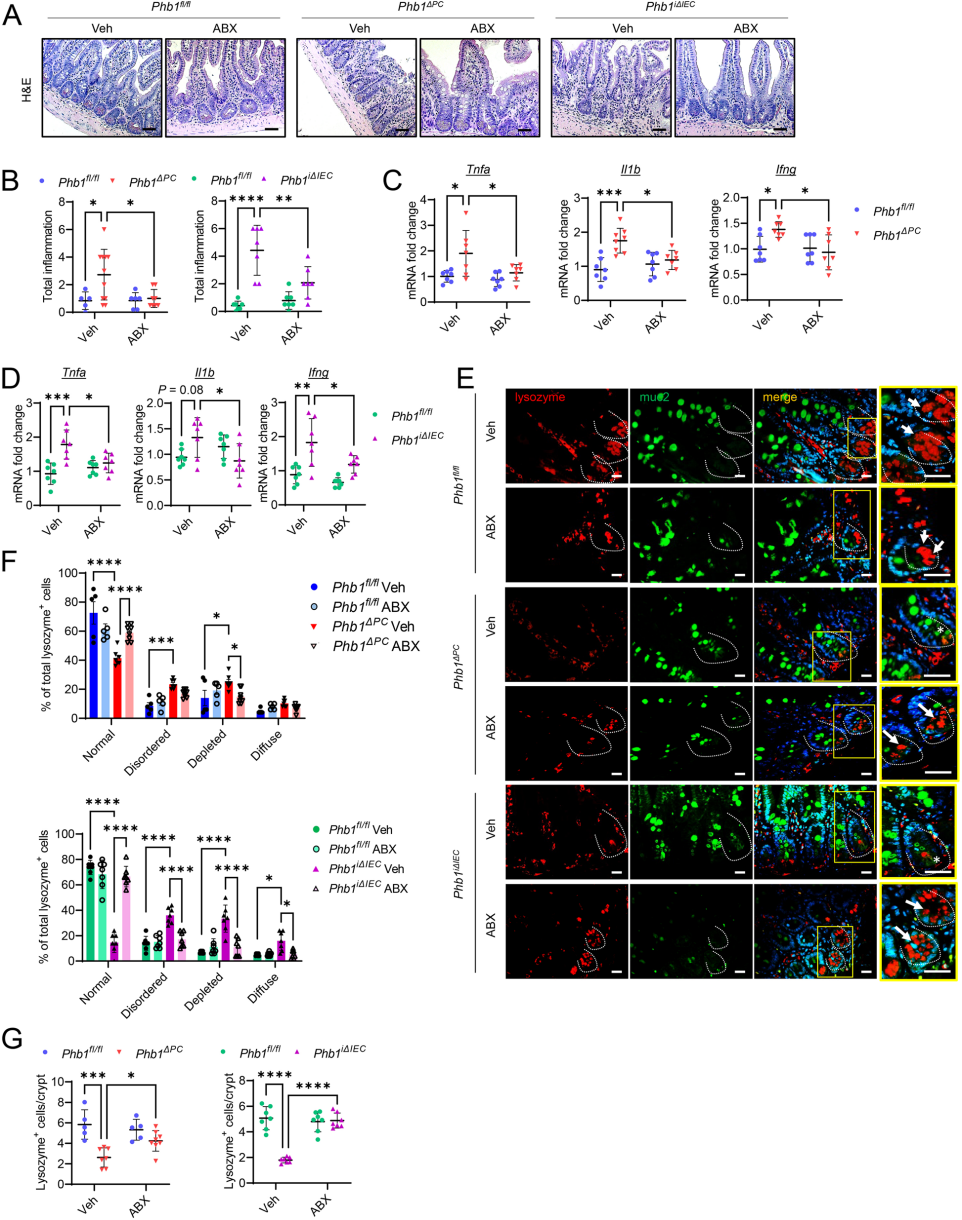
Elimination of gut microbiota by broad-spectrum antibiotics (ABX) ameliorates ileal inflammation and restores Paneth cell health in *Phb1*-deficient mice. **A** H&E staining of the ileum. Bar = 100 μm. **B** Histological inflammation scoring of H&E-stained ileal sections. *n* = 8–11 *Phb1*^*ΔPC*^ or 5–6 *Phb1*^*fl/fl*^ littermates; *n* = 7 *Phb1*^*iΔIEC*^ or 7 *Phb1*^*fl/fl*^ littermates. **C** mRNA quantification in the ileum of *Phb1*^*ΔPC*^ or *Phb1*^*fl/fl*^ littermates. *n* = 7 per genotype. **D** mRNA quantification in the ileum of *Phb1*^*iΔIEC*^ or *Phb1*^*fl/fl*^ littermates. *n* = 7 per genotype. **E** Immunofluorescent-staining for lysozyme (red), muc2 (green), and DAPI (nucleus, blue) in ileal crypts (dashed line). Arrows denote Paneth cells with normal lysozyme packaging into granules. Star denotes lysozyme/muc2 colocalization in the crypt base. Bar = 50 μm. **F** Paneth cell lysozyme allocation patterns across 50 ileal crypts. *n* = 7–8 each group. **G** Average number of lysozyme^+^ cells per crypt per mouse. A minimum of 50 crypts per mouse were quantitated. *n* = 7–8 *Phb1*^*ΔPC*^ or 5 *Phb1*^*fl/fl*^ littermates; *n* = 7 *Phb1*^*iΔIEC*^ or 7 *Phb1*.^*fl/fl*^ littermates. ABX, antibiotics; H&E, hematoxylin & eosin; Ifng, interferon gamma; Il1b, interleukin 1 beta; muc2, mucin2; Tnfa, tumor necrosis factor alpha; Veh, vehicle. Results are presented as individual mice ± SD. *P < 0.05, **P < 0.01, ***P < 0.001, and ****P < 0.0001

In our previous study, we demonstrated that mice with deletion of *Phb1* in IECs (*Phb1*^*i∆IEC*^ mice), including Paneth cells, manifested spontaneous ileitis by 20 weeks of age that was preceded by mitochondrial dysfunction in all epithelial cells, gut microbiota dysbiosis, and early abnormalities in Paneth cells [20]. Deletion of *Phb1* specifically in Paneth cells (*Phb1*^*∆PC*^ mice) was sufficient to induce Paneth cell defects and to cause ileitis in mice [20]. To understand whether gut microbiota is necessary for the development of spontaneous ileitis driven by mitochondrial dysfunction, broad-spectrum antibiotics (ABX) were administered to *Phb1*^*ΔPC*^ and *Phb1*^*iΔIEC*^ mice and their *Phb1*^*fl/fl*^ littermates in their drinking water from 16 to 20 weeks of age. Depleting gut microbiota by ABX (Figure S1A) protected *Phb1*-deficient mice from spontaneous ileitis measured histologically (Fig. 1A, B) and from proinflammatory cytokine expression upregulated in these mouse models (Fig. 1C, D) [20]. Additionally, using scoring criteria previously described [25], ABX ameliorated abnormal Paneth cell granularity (disordered, depleted, and diffuse staining) in *Phb1*-deficient mice showing lysozyme properly packaged into secretory granules (Fig. 1E, F). Paneth cell abnormalities in *Phb1*^*ΔPC*^ and *Phb1*^*iΔIEC*^ mice, including the manifestation of intermediate Paneth/goblet cells marked by lysozyme^+^/muc2^+^ cells (Fig. 1E), loss of lysozyme^+^ cells (Fig. 1G), and increased alcian blue^+^ cells in the crypt base (Figure S1B, C), were prevented by ABX. These results suggest that mitochondrial dysfunction driven by the loss of PHB1 in IECs or specifically in Paneth cells is not sufficient to cause intestinal inflammation, but when coupled with gut microbiota interaction can trigger disease.

### Phb1^ΔPC^ mice fail to demonstrate significant alterations in the ileal microbial communities

**Fig. 2:**
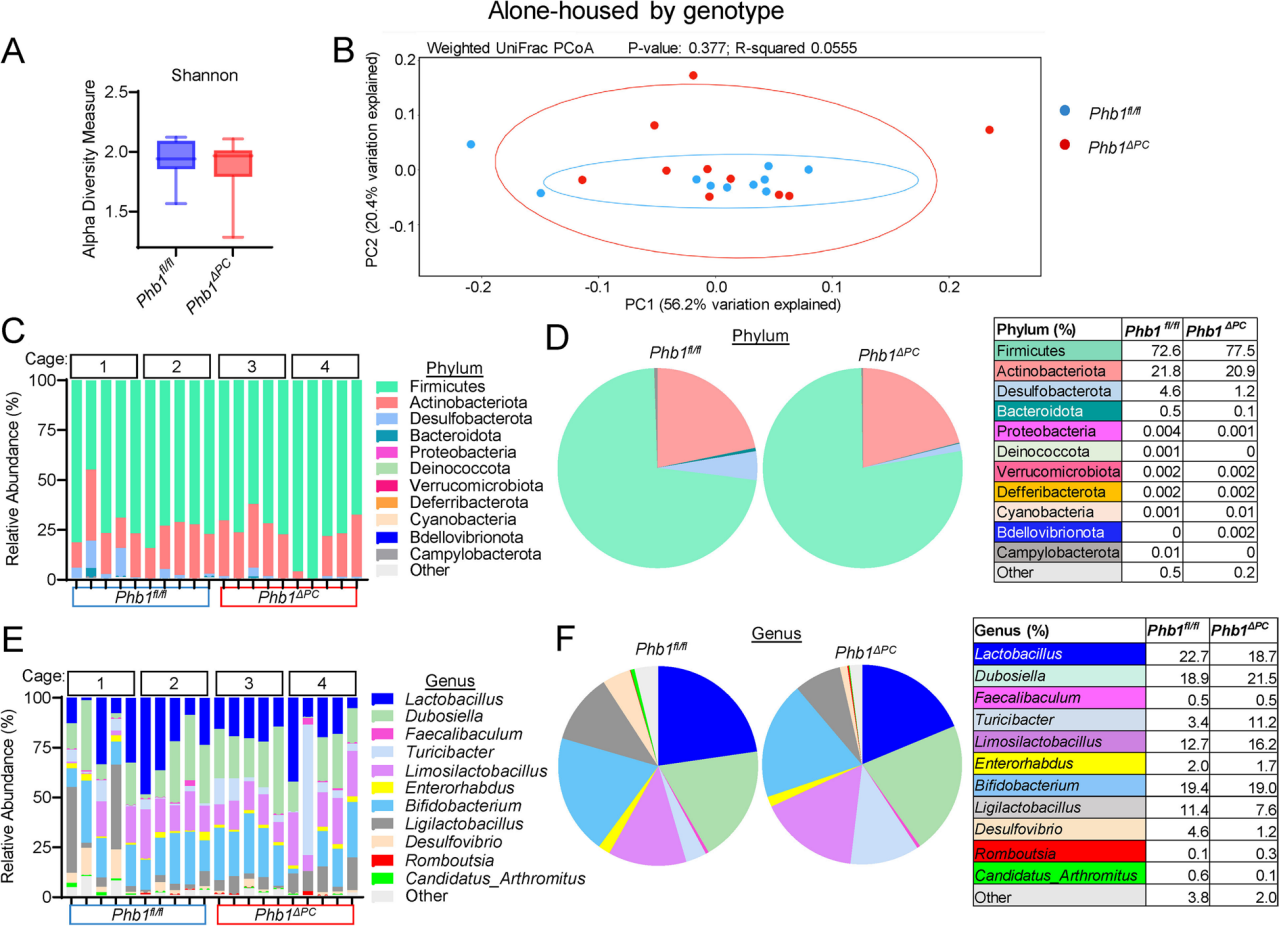
*Phb1*^*ΔPC*^ mice exhibit similar gut microbiota composition as *Phb1*^*fl/fl*^ littermates when alone-housed by genotype. 16S RNA sequencing of ileal luminal contents of alone-housed *Phb1*^*fl/fl*^ and *Phb1*^*ΔPC*^ mice. **A** Alpha diversity measure by Shannon index. **B** Weighted UniFrac PCoA plot of 16S rRNA gene sequences. **C**, **D** Relative abundance of bacteria at phylum level in individual mice (**C**) and combined mice by genotype (**D**). **E**, **F** Relative abundance of bacteria at genus level in individual mice (**E**) and combined mice by genotype (**F**). *n* = 10 for each genotype

**Fig. 3:**
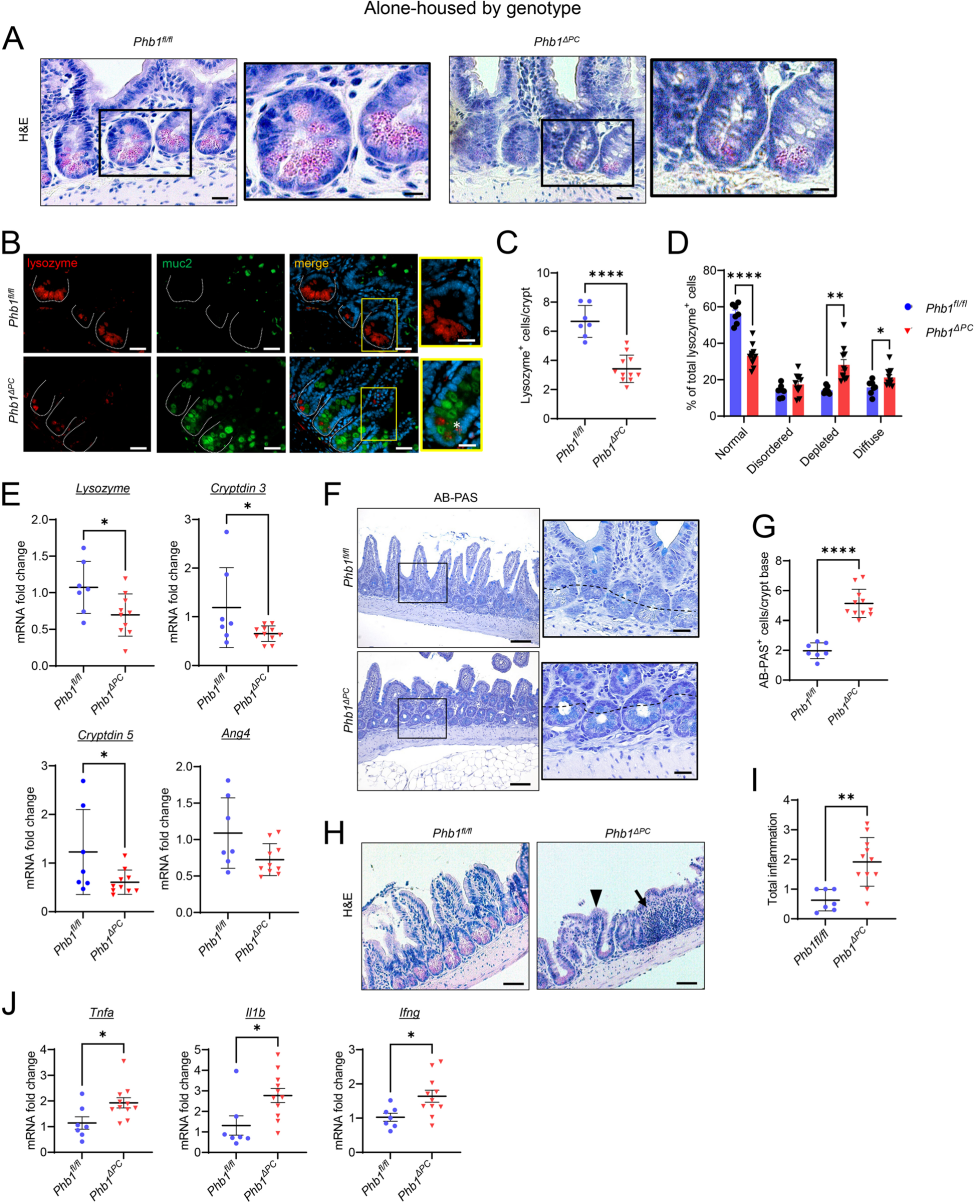
Alone-housed *Phb1*^*ΔPC*^ mice manifest Paneth cell defects and spontaneous ileitis. **A** H&E staining showing Paneth cells (pink granules) in the ileal crypts. Bar = 150 μm; boxed pullout bar = 75 μm. **B** Immunofluorescent-staining for lysozyme (red), muc2 (green), and DAPI (nucleus, blue) in ileal crypts (dashed line). Star denotes lysozyme/muc2 colocalization in the crypt base. Bar = 150 μm; boxed pullout bar = 75 μm. **C** Average number of lysozyme^+^ cells per crypt per mouse. A minimum of 50 crypts per mouse were quantitated. **D** Paneth cell lysozyme allocation patterns. A minimum of 50 crypts per mouse were quantitated. **E** mRNA quantification in the ileum. One outlier was identified by ROUT test (*Q* = 1%) and was removed from *Phb1*^*ΔPC*^* lysozyme*, *cryptdin5*, and *ang4*). **F** AB-PAS staining of the ileum. Bar = 100 µm. The dashed line denotes the crypt base. **G** The number of AB-PAS^+^ cells/crypt base across 50 crypts. **H** Histological inflammation scoring of the ileum. The arrow denotes immune cell infiltration, and the arrowhead denotes villus blunting. Bar = 100 µm. **I** Histological inflammation scoring of H&E-stained ileal sections. **J** mRNA quantification in the ileum. *n* = 7 *Phb1*^*fl/fl*^ or 11 *Phb1*^*Δ*^.^*PC*^ mice. AB-PAS, alcian blue-periodic acid Schiff; Ang4, angiogenin 4; H&E, hematoxylin & eosin; Ifng, interferon gamma; Il1b, interleukin 1 beta; muc2, mucin2; Tnfa, tumor necrosis factor alpha. Results are presented as individual mice ± SD. *P < 0.05, **P < 0.01, and ****P < 0.0001

Our previous study demonstrated that *Phb1*^*iΔIEC*^ mice exhibited microbiota dysbiosis including decreased diversity by Shannon index and decreased abundance of specific taxa such as *Blautia*, *Roseburia*, *Oscillibacter*, and *Coprococcus* [20]. To evaluate changes in the gut microbiota composition of *Phb1*^*ΔPC*^ mice, 16S rRNA sequencing was performed on luminal ileal contents from *Phb1*^*fl/fl*^ (control) and *Phb1*^*ΔPC*^ littermate mice alone-housed by genotype or co-housed across genotypes at weaning to 20 weeks of age. Our data does not indicate any difference in alpha diversity by Shannon index, species beta diversity by weighted UniFrac PCoA analysis, nor phylum and genus composition between *Phb1*^*ΔPC*^ and *Phb1*^*fl/fl*^ ileal microbiotas during alone-housing (Fig. 2) or co-housing (Figure S2). These results suggest that *Phb1*^*ΔPC*^ mice fail to demonstrate significant alterations in the ileal microbial communities. Despite this, both alone-housed (Fig. 3) and co-housed *Phb1*^*ΔPC*^ mice (Figure S3) exhibited Paneth cell defects and ileitis. However, co-housing eliminated the increased mucin staining in the crypt base of *Phb1*^*ΔPC*^ mice (Figure S3F). Unlike our results shown here in *Phb1*^*ΔPC*^ mice, our previous study characterizing ileitis in alone-housed *Phb1*^*i∆IEC*^ mice revealed gut microbiota alterations [20]. Given this, we next assessed the phenotype of *Phb1*^*i∆IEC*^ mice in the presence of control microbiota during co-housing with respective *Phb1*^*fl/fl*^ littermates. Co-housed *Phb1*^*i∆IEC*^ mice exhibited Paneth cell defects and spontaneous ileitis (Figure S4), suggesting the dominance of this phenotype even in the presence of healthy microbiota during co-housing with *Phb1*^*fl/fl*^ littermates. Similar to co-housed *Phb1*^*ΔPC*^ mice, co-housed *Phb1*^*i∆IEC*^ mice demonstrated normal mucin staining (Figure S4F), suggesting a normalizing effect of healthy microbiota on this alteration during host mitochondrial dysfunction.

### Wild-type germ-free mice conventionalized with Phb1^i∆IEC^ ileal microbiota do not manifest ileitis

**Fig. 4:**
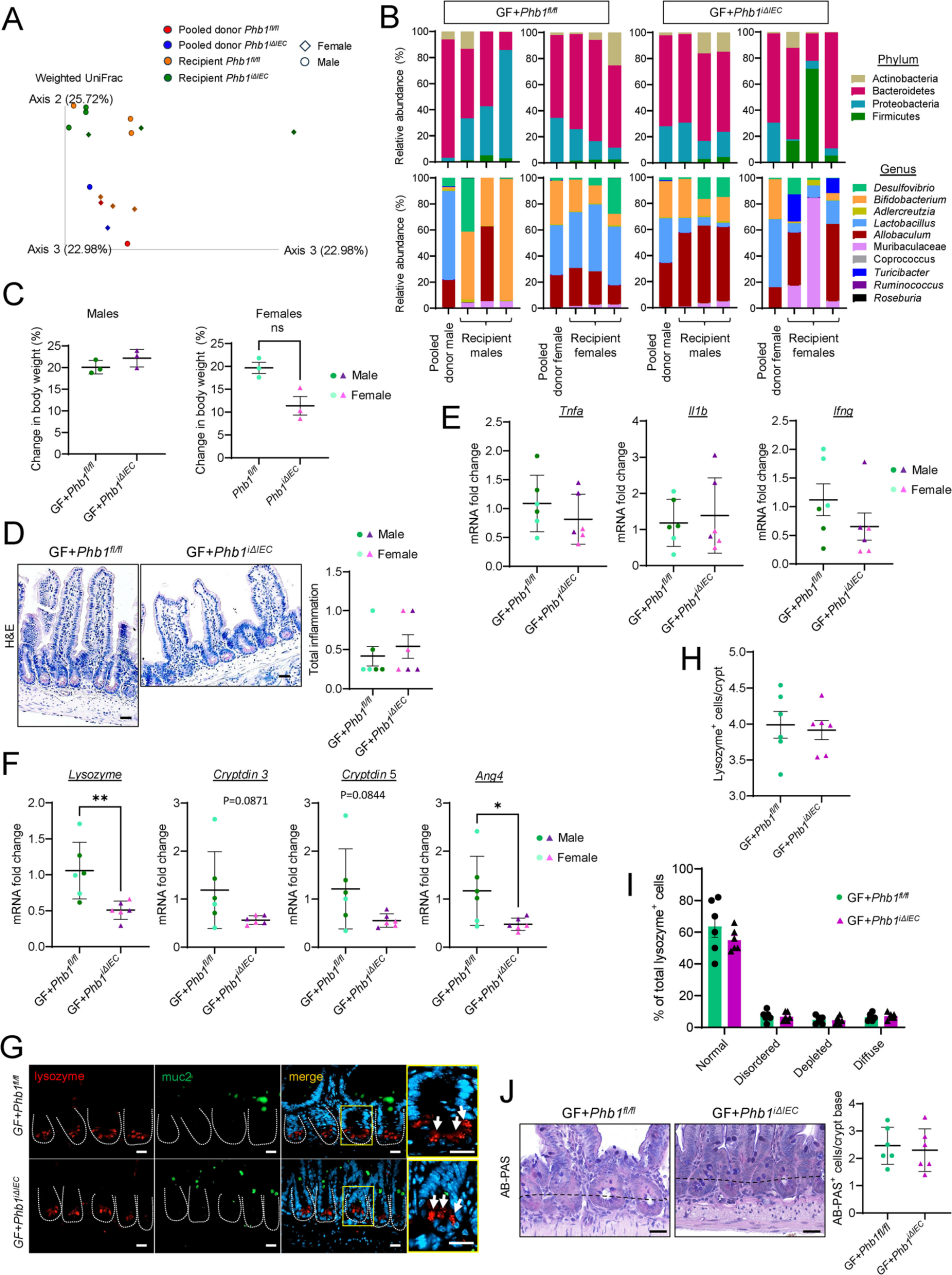
Wild-type germ-free mice conventionalized with *Phb1*^*i∆IEC*^ dysbiotic ileal microbiota exhibit Paneth cell defects but do not manifest ileitis. **A** Weighted UniFrac PCoA plot of 16S rRNA sequencing of ileal stools from the donor (*Phb1*^*fl/f*^ and *Phb1*^*iΔIEC*^ mice pooled by sex) and the recipient (GF + *Phb1*^*fl/f*^ and GF + *Phb1*^*iΔIEC*^) mice. **B** Relative abundance of bacteria at phylum and genus level. **C** Body weight of conventionalized germ-free mice at 20 weeks of age. **D** H&E-stained ileum and histology scoring. Bar = 100 μm. **E** mRNA fold change by qRT-PCR of markers of inflammation in the ileum. **F** mRNA expression of AMPs in the ileum. **G** Immunofluorescent staining for lysozyme (red), muc2 (green), and DAPI (nucleus, blue) in ileal crypts (dashed line). Arrows denote Paneth cells with normal Lysozyme packaging into granules. Bar = 50 μm. **H** Average number of lysozyme^+^ cells per crypt per mouse. **I** Paneth cell lysozyme allocation patterns. **J** AB-PAS staining of the ileum and number of AB-PAS^+^ cells/crypt base across 50 crypts. Bar = 100 µm. The dashed line denotes the crypt base. *n* = 6 GF + *Phb1*^*fl/fl*^ or GF + *Phb1*^*iΔIEC*^ recipient mice, *n* = 4 *Phb1*^*fl/fl*^ and *Phb1*.^*iΔIEC*^ pooled donor mice. AB-PAS, alcian blue-periodic acid Schiff; Ang4, angiogenin 4; GF, germ free; H&E, hematoxylin & eosin; Ifng, interferon gamma; Il1b, interleukin 1 beta; muc2, mucin2; Tnfa, tumor necrosis factor alpha. Results are presented as individual mice ± SD. *P < 0.05 and **P < 0.01

To determine whether the established *Phb1*^*i∆IEC*^ dysbiotic microbiota transfers phenotype in the absence of genetic alterations, 8-week-old wild-type germ-free (GF) mice were conventionalized with specific pathogen-free (SPF) ileal microbiota from 20-week-old *Phb1*^*fl/fl*^ or *Phb1*^*i∆IEC*^ mice and were maintained until sacrifice in the gnotobiotic facility until 20 weeks of age (12 weeks after colonization). Ileal microbiota were used as donor material since our mouse models of *Phb1* deletion develop ileitis without affecting the colon [20]. Since GF mice are routinely conventionalized with cecal contents but we are unaware of any reports using ileal contents, 16S rRNA sequencing on ileal contents of GF recipient mice and donor ileal luminal contents was performed to evaluate transferability of gut microbiota (Fig. 4A, B). GF mice conventionalized with *Phb1*^*i∆IEC*^ ileal microbiota did not exhibit any overt phenotype including body weight gain by 20 weeks of age (Fig. 4C). Recipient GF mice did not develop ileitis regardless of the donor genotype (Fig. 4D, E). However, GF mice conventionalized with *Phb1*^*i∆IEC*^ ileal microbiota exhibited decreased AMP expression (Fig. 4F), but other Paneth cell alterations were not noted (Fig. 4G–J). These results in wild-type GF mice suggest that *Phb1*^*i∆IEC*^ dysbiotic ileal microbiota is not sufficient to induce ileitis by 12 weeks after colonization in the absence of host genetic alteration.

### Butyrate supplementation prevents ileitis and Paneth cell defects in Phb1-deficient mice

**Fig. 5:**
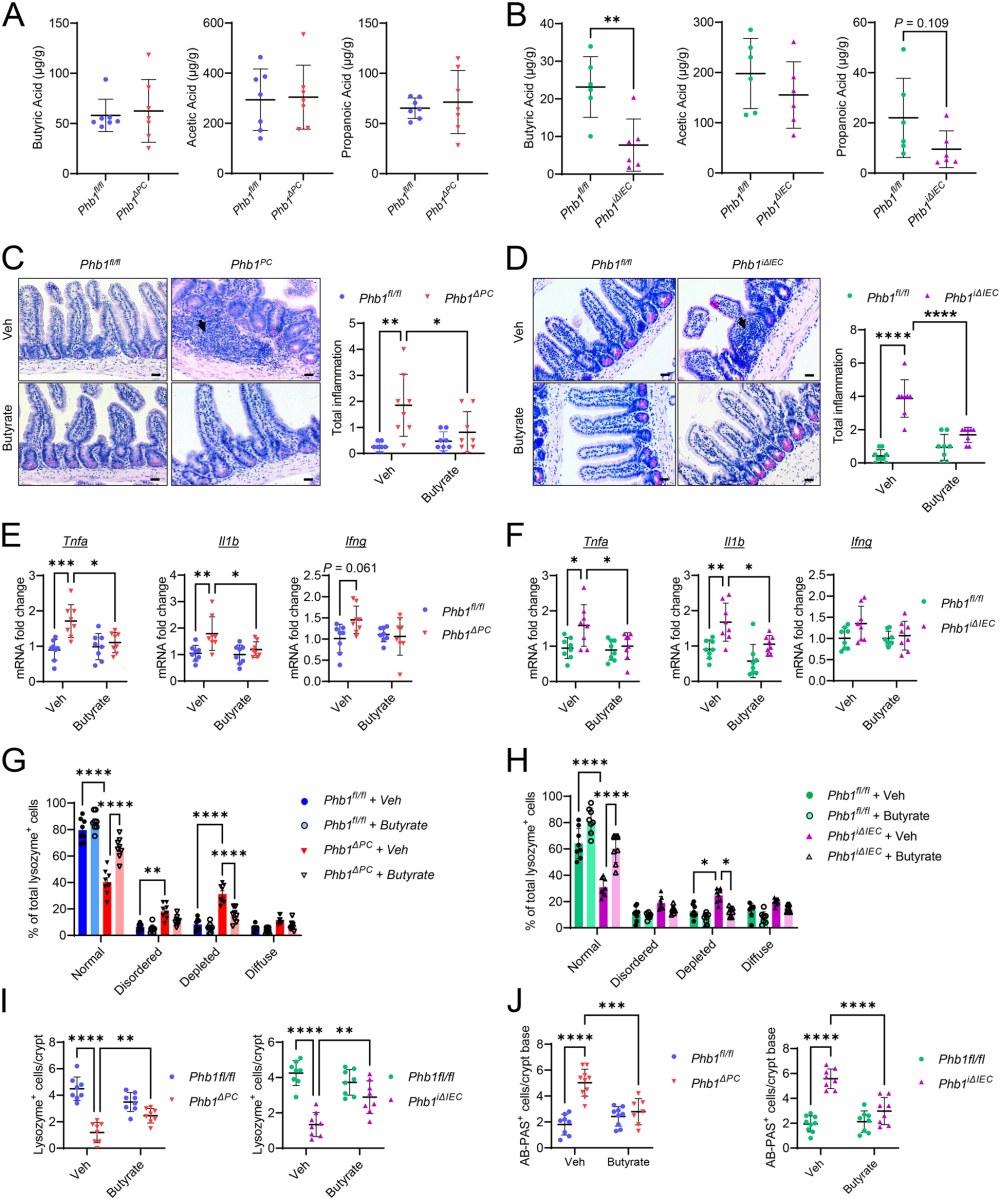
Butyrate supplementation prevents ileitis and Paneth cell defects in *Phb1*-deficient mice. **A** SCFAs in luminal ileal feces of *Phb1*^*ΔPC*^ mice and *Phb1*^*fl/fl*^ littermates. *n* = 7 for each genotype. **B** SCFAs in luminal ileal feces of *Phb1*^*iΔIEC*^ mice and *Phb1*^*fl/fl*^ littermates. *n* = 6 for each genotype. **C**, **D** H&E staining of the ileum and histological inflammation scoring in **C** *Phb1*^*ΔPC*^ mice and **D **Phb1^*iΔIEC*^ mice. Arrows denote immune cell infiltration. Bar = 50 μm.* n* = 8 for each genotype. **E** mRNA quantification in the ileum of *Phb1*^*ΔPC*^ or *Phb1*^*fl/fl*^ littermates. *n* = 8 per genotype. **F** mRNA quantification in the ileum of *Phb1*^*iΔIEC*^ or *Phb1*^*fl/fl*^ littermates. *n* = 8 per genotype. **G**, **H** Paneth cell lysozyme allocation patterns. *n* = 8 per genotype. **I** Average number of lysozyme^+^ cells across 50 crypts. *n* = 8 per genotype. **J** Number of AB-PAS.^+^ cells/crypt base across 50 crypts. *n* = 8 per genotype. AB-PAS, alcian blue-periodic acid Schiff; Ang4, angiogenin 4; Ifng, interferon-gamma; Il1b, interleukin 1 beta; Tnfa, tumor necrosis factor alpha; Veh, vehicle. Results are presented as individual mice ± SD. *P < 0.05, **P < 0.01, ***P < 0.001, and ****P < 0.0001

To better understand the effect of host epithelial mitochondrial dysfunction on gut microbiota, the 3 most abundant SCFAs, butyric acid, acetic acid, and propanoic acid, were measured in ileal feces of *Phb1*^*ΔPC*^ or *Phb1*^*iΔIEC*^ mice and respective *Phb1*^*fl/fl*^ littermates (alone-housed by genotype) as an indicator of microbiota-produced metabolites. *Phb1*^*ΔPC*^ mice exhibited no change in these SCFAs (Fig. 5A). Butyric acid, but not acetic acid or propanoic acid, was significantly decreased in the ileal feces of *Phb1*^*iΔIEC*^ mice (Fig. 5B). To elucidate the effect of butyrate supplementation on Paneth cell health and ileal inflammation driven by *Phb1* deletion, *Phb1*^*fl/fl*^, *Phb1*^*ΔPC*^, and *Phb1*^*iΔIEC*^ mice were treated with 0.5-mM sodium butyrate in drinking water from 16 to 20 weeks of age. Butyrate treatment decreased the severity of ileal inflammation (Fig. 5C–F), restored lysozyme packaging into granules (Figs. 5G, H, S5A, B), and restored numbers of lysozyme^+^ cells in *Phb1*^*ΔPC*^ and *Phb1*^*iΔIEC*^ mice (Fig. 5I). Butyrate supplementation ameliorated the development of intermediate Paneth/goblet cells as indicated by lacking lysozyme^+^/muc2^+^ co-staining in butyrate-treated *Phb1*^*ΔPC*^ and *Phb1*^*iΔIEC*^ mice (Figure S5A, B) and numbers of alcian blue^+^ cells in the crypt base matching *Phb1*^*fl/fl*^ mice (Figs. 5J, S5C, D). Taken together, these data demonstrate the therapeutic potential of butyrate on Paneth cell abnormalities and ileitis driven by epithelial mitochondrial dysfunction during *Phb1* deletion.

### Butyrate protects Phb1-deficient ileal enteroids from dysbiotic ileal microbiota-induced death

**Fig. 6:**
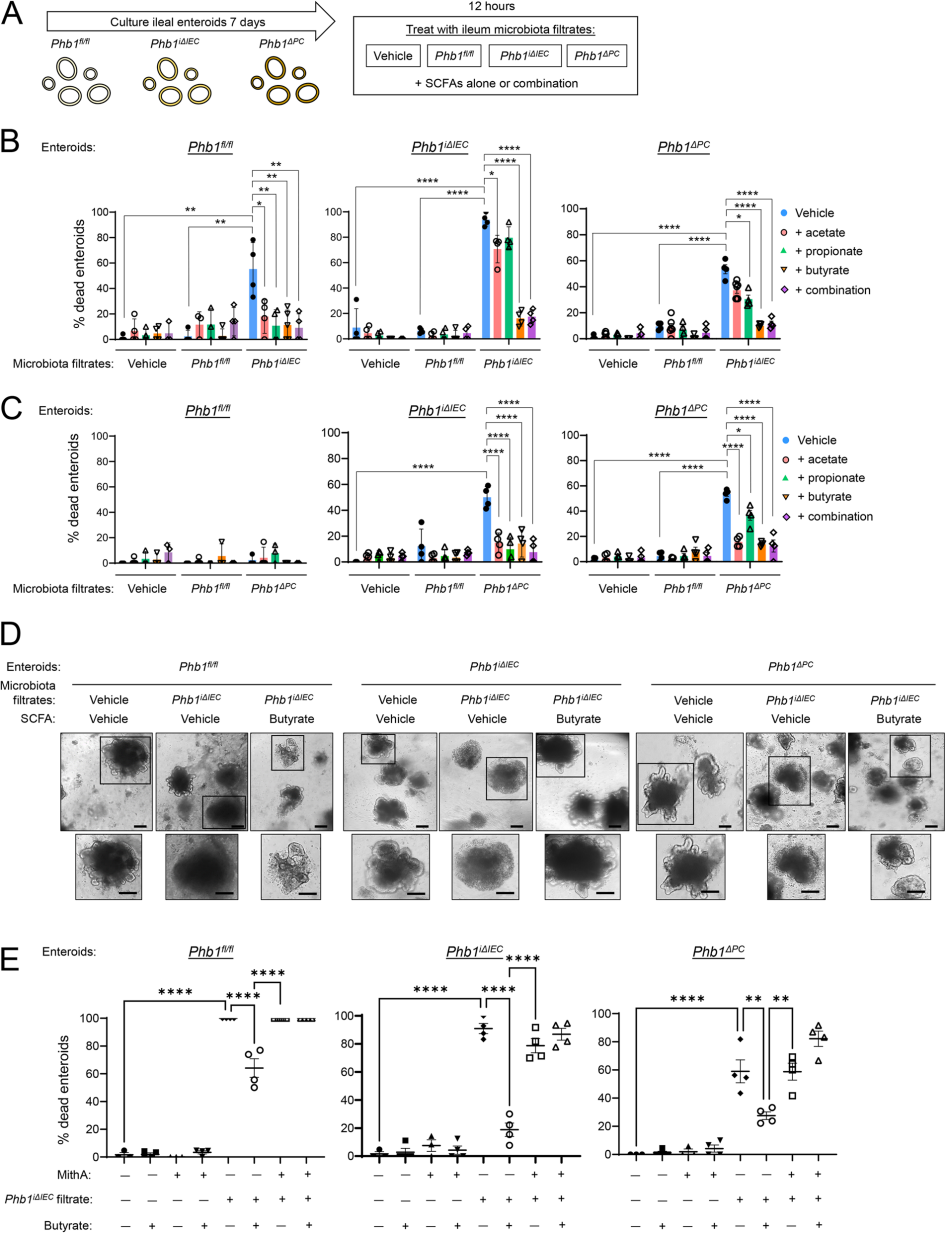
Butyrate protects *Phb1*-deficient ileal enteroids from dysbiotic microbiota-induced death. **A** Schematic of the experimental protocol of ileal enteroid treatment. Enteroids were cultured from ileal crypts of *Phb1*^*fl/fl*^, *Phb1*^*iΔIEC*^, or *Phb1*^*ΔPC*^ mice for 7 days and treated for 12 h with vehicle or 200 μg/ml ileal luminal feces filtrate obtained from 20-week-old *Phb1*^*fl/fl*^, *Phb1*^*iΔIEC*^, or *Phb1*^*ΔPC*^ mice. Concurrent with microbiota treatment, enteroids were treated with vehicle or 0.5 mM SCFAs alone or in combination. **B**, **C** Enteroid death as measured by morphological change during treatment with **B** *Phb1*^*fl/fl*^ or *Phb1*^*iΔIEC*^ microbiota filtrate or **C** *Phb1*^*fl/fl*^ or *Phb1*^*Δ*^^*PC*^ microbiota filtrate. **D** Representative enteroid images. Bar = 100 µm. **E** Enteroid death by morphological change. Enteroids were treated with 1 µM Mithramycin A for 1 h prior to microbiota filtrate/butyrate treatment as in **A**. *n* = 4–6 for all treatments. MithA, Mithramycin A. Results are presented as individual mice ± SD. *P < 0.05, **P < 0.01, and ****P < 0.0001

To determine microbiota-induced epithelial-intrinsic effects, enteroids were cultured from ileal crypts of 8-week-old *Phb1*^*fl/fl*^, *Phb1*^*iΔIEC*^, and *Phb1*^*ΔPC*^ mice for 7 days followed by treatment with ileal fecal filtrate obtained from 20-week-old *Phb1*^*iΔIEC*^ or *Phb1*^*ΔPC*^ mice or respective *Phb1*^*fl/fl*^ mice (control microbiota) (Fig. 6A). After 12 h of treatment, microbial filtrates derived from *Phb1*^*iΔIEC*^ mice induced ~ 90% death of *Phb1*^*iΔIEC*^ enteroids and ~ 50% death of *Phb1*^*fl/fl*^ and *Phb1*^*ΔPC*^ enteroids (Fig. 6B). In comparison, microbial filtrates derived from *Phb1*^*fl/fl*^ mice did not induce enteroid death of any genotype (Fig. 6B, C). In *Phb1*^*fl/fl*^ enteroids, microbial filtrates derived from *Phb1*^*ΔPC*^ mice did not induce death, similar to control *Phb1*^*fl/fl*^ microbial filtrates and vehicle, but in *Phb1*^*iΔIEC*^ and *Phb1*^*ΔPC*^ enteroids elicited ~ 50% death (Fig. 6C). The response of enteroids to microbial filtrates was confirmed by immunoblotting for Cleaved Caspase 3 (CC3), a marker of apoptosis. The amount of CC3 was markedly increased in *Phb1*^*ΔPC*^ and *Phb1*^*iΔIEC*^ enteroids treated with microbial filtrates (Figure S6A). These results suggest an important interaction between impaired intestinal epithelial mitochondrial health and the environmental trigger of microbiota on epithelial homeostasis. The most severe response was noted in enteroids lacking PHB1 throughout the epithelium (*Phb1*^*iΔIEC*^ enteroids) coupled with interaction with *Phb1*^*iΔIEC*^ microbial filtrates.

In colon epithelial cells, SCFAs are essential in glucose metabolism, fatty acid oxidation, mitochondrial biogenesis, and oxidative phosphorylation [33, 35–37]. The role of SCFAs in IEC health has been well-studied in the colon but is less understood in the ileum where the concentration of SCFAs is ~ fivefold lower compared to the colon [8]. SCFAs are rapidly absorbed in the human ileum [38] and are required for IEC homeostasis, including proliferation and migration along the crypt-villus axis [9]. In ileal enteroid models, SCFAs were shown to induce proliferation and inhibit HDAC activity, driving changes in gene transcription [39]. To determine the effect of SCFAs on ileal enteroid viability, ileal enteroids from *Phb1*^*fl/fl*^, *Phb1*^*ΔPC*^, and *Phb1*^*iΔIEC*^ mice were treated with butyrate, acetate, propionate (the conjugate base forms of butyric, acetic, or propanoic acids, respectively), or all 3 SCFAs in combination concurrent with ileal microbial filtrate treatment (Fig. 6A). With regard to death induced by *Phb1*^*iΔIEC*^ microbial lysate, all SCFAs were remarkably protective in *Phb1*^*fl/fl*^ enteroids but only butyrate provided maximal protection against the death of *Phb1*^*iΔIEC*^ or *Phb1*^*ΔPC*^ enteroids (Fig. 6B, D). Apoptosis as measured by CC3 was prevented by butyrate in *Phb1*^*ΔPC*^ and *Phb1*^*iΔIEC*^ enteroids treated with microbial filtrates from *Phb1*^*iΔIEC*^ mice (Figure S5A). The combination of butyrate, acetate, and propionate behaved similar to that of butyrate alone, ameliorating *Phb1*^*iΔIEC*^ or *Phb1*^*ΔPC*^ enteroid death during exposure to *Phb1*^*iΔIEC*^ microbial filtrates (Fig. 6B). When exposed to microbial filtrates from *Phb1*^*ΔPC*^ mice, all SCFAs elicited protection against death (Fig. 6C). Taken together, these data demonstrate enhanced enteroid viability by butyrate, acetate, and propionate during Paneth cell-specific mitochondrial impairment and microbiota challenge. Butyrate exhibits maximal protection against mitochondrial dysfunction affecting all IECs during interaction with gut microbial filtrates.

### Sp1 contributes to butyrate-induced protection of intestinal epithelium

SCFAs elicit many responses in host IECs including energy metabolism, alteration of HDAC activity, dampening the mucosal immune response, and promoting epithelial barrier integrity [33, 35–37]. To further understand butyrate protection in IECs during mitochondrial dysfunction driven by *Phb1* deletion, oxygen consumption rate and mitochondrial-derived superoxide were measured in ileal enteroids derived from *Phb1*^*fl/fl*^ and *Phb1*^*iΔIEC*^ mice as a measure of energy metabolism via oxidative phosphorylation. Superoxide is a reactive oxygen species (ROS) produced as a byproduct of mitochondrial electron transport chain (ETC) activity; the level of mitochondrial-derived superoxide is increased when ETC activity is suboptimal and electrons aberrantly leak to molecular oxygen [40]. As a positive control, enteroids were treated with Antimycin A (an ETC inhibitor and ROS producer). As expected, Antimycin A decreased the level of available oxygen with a concurrent increase in mitochondrial superoxide in *Phb1*^*fl/fl*^ enteroids (Figure S6B and C), suggesting available oxygen is converted to superoxide during Antimycin A inhibition of the ETC. *Phb1*^*iΔIEC*^ enteroids, with or without Antimycin A treatment, exhibited decreased available oxygen and increased mitochondrial superoxide similar to that of *Phb1*^*fl/fl*^ Antimycin A-treated enteroids (Figure S6B and C), suggesting enhanced ROS production during loss of PHB1, as we previously reported [20]. Interestingly, butyrate ameliorated increased mitochondrial superoxide in *Phb1*^*iΔIEC*^ enteroids (Figure S6C). Control microbial filtrates did not affect oxygen consumption or mitochondrial superoxide levels in *Phb1*^*iΔIEC*^ enteroids, whereas *Phb1*^*iΔIEC*^ microbial filtrates greatly exacerbated mitochondrial superoxide production and increased oxygen availability, which could not be rescued by butyrate supplementation (Figure S6B and C). These results suggest that butyrate exerts protection to inhibit mitochondrial superoxide production during *Phb1* deletion alone, but upon exacerbation with *Phb1*^*iΔIEC*^ microbial filtrates, butyrate does not dampen ROS by this insult. Therefore, butyrate provides protection from IEC mitochondrial dysfunction during interaction with microbiota through mechanisms largely other than mitochondrial energy metabolism.

Inhibition of HDACs is a well-established role of butyrate in IECs [41]. Since cooperation at gene promoters between Sp1, HDACs, and transcriptional co-activators such as p300 is central to butyrate action on gene transcription [42, 43], we next utilized Mithramycin A, a selective Sp1 inhibitor, to prevent Sp1-associated transcriptional changes during butyrate treatment. The addition of Mithramycin A prevented butyrate protection against cell death induced by *Phb1*^*iΔIEC*^ microbiota in *Phb1*^*fl/fl*^, *Phb1*^*iΔIEC*^, and *Phb1*^*ΔPC*^ enteroids (Fig. 6E). These results suggest a possible role of Sp1-regulated gene transcription by butyrate in protection against microbiota shaped by host mitochondrial dysfunction.

## Discussion

As the interface between the inside and the outside world, the intestinal epithelium serves as a hub of bidirectional cross-talk between host and luminal microbiota. The ileal microbiome is a complex community, although with less abundance and diversity (~ 10^6^ bacterial counts/ml) than that of the colon (~ 10^10^ bacterial counts/ml) [44]. At the phylum level, Firmicutes and Proteobacteria dominate the small intestine, whereas Bacteroidetes is increased in the colon [45]. Patients with Crohn’s disease have gut microbiome dysbiosis compared with healthy controls, including decreased diversity and alterations in bacterial composition [3, 4]. A higher number of adherent ileal mucosal-associated bacteria are also noted in patients with Crohn’s disease, including decreased Firmicutes and increased Proteobacteria such as *Escherichia coli* [46, 47]. Microbial dysbiosis in Crohn’s disease is thought to result from the development of inflammation and to perpetuate disease. Microbiota and host signaling have been shown to involve the mitochondria within the intestinal epithelium in mouse models of colitis [48]. Our current research delves deeper into understanding the impact of epithelial mitochondria on small intestinal health. We show an important role of host IEC mitochondrial function in influencing intestinal microbiota metabolites during the development of ileitis.

To generate mouse models of mitochondrial dysfunction in IECs or specifically in Paneth cells, we targeted the deletion of mitochondrial chaperone protein PHB1 which is decreased in mucosal biopsies from IBD patients [21, 23]. Unlike *Phb1*^*i∆IEC*^ mice that exhibited altered gut microbiota composition [20], we show here that Paneth cell-specific mitochondrial dysfunction in *Phb1*^*∆PC*^ mice under co-housing with control *Phb1*^*fl/fl*^ littermates or alone-housing by genotype did not alter ileal microbial communities or SCFA levels despite the manifestation of Paneth cell defects and ileitis. This suggests that deletion of PHB1 throughout the intestinal epithelium drives microbiota dysbiosis, whereas deletion of PHB1 limited to Paneth cells is not sufficient to alter microbiota composition or SCFAs. Perhaps *Phb1*^*i∆IEC*^ mice manifest microbiota dysbiosis since, in addition to Paneth cells, other epithelial cells including goblet cells and crypt base columnar stem cells exhibited alterations that may drive a more robust luminal phenotype [20]. Despite similar microbial composition in *Phb1*^*ΔPC*^ as *Phb1*^*fl/fl*^ mice, our results using broad-spectrum antibiotics treatment demonstrated a necessary role of the gut microbiota in the development of spontaneous ileitis and Paneth cell defects in both *Phb1*^*ΔPC*^ and *Phb1*^*iΔIEC*^ mice. This suggests that IEC or Paneth cell mitochondrial dysfunction driven by the loss of PHB1 is not sufficient to cause intestinal inflammation, but requires interaction with gut microbiota for the development of disease. We further show that ileal microbial filtrates from *Phb1*-deficient mice, but not from control *Phb1*^*fl/fl*^ mice, induced the death of ileal enteroids derived from *Phb1*-deficient mice. Collectively, these results suggest an important interaction between impaired epithelial mitochondrial health and microbiota driving loss of intestinal homeostasis.

We next determined whether ileal microbial composition could be transferred to GF mice and induce Paneth cell defects or ileitis in wild-type mice without mitochondrial impairment. GF mice colonized with ileal microbiota from dysbiotic *Phb1*^*iΔIEC*^ mice did not develop Paneth cell defects or ileitis. This suggests that the dysbiotic ileal microbiota from *Phb1*-deficient mice cannot independently induce intestinal inflammation without host mitochondrial dysfunction. Interestingly, a previous study using GF mice colonized with dysbiotic cecal microbiota of TNF^∆ARE^ mice, which develop spontaneous ileitis due to upregulation of TNFα, demonstrated an induction of ileitis after colonization that was preceded by the loss of Paneth cell function [49]. Of note, our study used ileal microbiota for colonization of GF mice since this was the region of the intestine affected during *Phb1* deletion versus cecal microbiota in the TNF^∆ARE^ study. Perhaps using cecal microbiota from *Phb1*-deficient mice would provide a more robust transfer into GF mice in future studies. Additionally, our results demonstrate some variability across the recipient GF mice with some engrafting better than others, which is consistent with previous studies in which transplant of fecal microbiomes into GF mice or antibiotic-depleted mice have a broad range between the donor microbiome and engrafted microbiome [50, 51]. This is a potential limitation that could contribute to the recipient GF mice not developing ileitis.

Microbiota-produced metabolites, including SCFAs, play an important role in maintaining gut homeostasis. Our results demonstrate that luminal butyrate was altered in the ileum of *Phb1*^*iΔIEC*^ mice and that supplementation of butyrate in *Phb1*^*iΔIEC*^ mice decreased the severity of Paneth cell abnormalities and ileal inflammation. Furthermore, in *Phb1*^*iΔIEC*^ enteroids, butyrate greatly enhanced viability during treatment with the microbiota of *Phb1*^*iΔIEC*^ mice, while acetate or propionate exhibited minimal protection. Collectively, these results suggest that mitochondrial dysfunction in all epithelial cells (*Phb1*^*iΔIEC*^) is associated with loss of butyrate contributing to epithelial impairment and ileitis. In contrast, all SCFAs tested (butyrate, propionate, and acetate) protected against enteroid death induced by *Phb1*^*ΔPC*^ dysbiotic microbiota. Furthermore, butyrate supplementation in *Phb1*^*ΔPC*^ mice was effective to decrease Paneth cell defects and ileitis despite these mice showing no loss of butyrate levels in ileal feces. We speculate that greater dependence on butyrate for protection in *Phb1*^*iΔIEC*^ mice is due to bulk mitochondrial impairment across all IECs including enterocytes, which in the colon have been shown to rely on microbial-derived butyrate for homeostatic function [17]. Our enteroid studies suggest a possible role of Sp1-regulated gene transcription by butyrate in epithelial cell protection against microbiota filtrates shaped by host mitochondrial dysfunction. Oral administration of butyrate in IBD patients, the most widely studied SCFA, has been shown to decrease inflammation and improve epithelial tight junction barrier integrity in some studies [52, 53]. Butyrate is currently sold as a dietary supplement in the USA. Previous studies have also shown that the administration of butyrate to animal models regulates inflammatory response, gut barrier integrity, and compensates dysbiotic gut microbiota [8, 54]. In addition, butyrate supplementation improves Paneth cell health and AMP secretion [55].

Taken together, we demonstrate that IEC mitochondrial dysfunction drives altered microbiota, and in turn, decreased butyrate expression in the ileum necessary for the development of ileitis. Restoration of SCFAs, especially butyrate, is a potential therapeutic option in Crohn’s disease patients harboring Paneth cell defects and epithelial mitochondrial impairment.

### Supplementary Information

**Additional file 1: Fig. S1.** Elimination of gut microbiota by broad-spectrum antibiotics (ABX) prevents the manifestation of crypt base AB-PAS+cells in Phb1 deficient mice. (A) Bacterial elimination by ABX shown by reads per sample by 16S rRNA sequencing. (B) AB-PAS staining of ileum. Bar = 100 μm. Dashed line denotes crypt base. (C) Number of AB-PAS+cells/crypt base across 50 crypts.AB-PAS, alcian blue-periodic acid Schiff. *n *= 7-13 each group. ****P< 0.0001. **Fig. S2.** Co-housed Phb1ΔPC mice exhibit similar ileal microbiota composition as Phb1fl/fl littermates.16S RNA sequencing of luminal ileal content of co-housed Phb1fl/fl and Phb1ΔPC mice. (A) Alpha diversity measure by Shannon index. (B) Weighted UniFrac PCoA plot of 16S rRNA gene sequences. (C-D) Relative abundance of bacteria at phylum level (C) in individual mice and (D) combined mice by genotype. (E-F) Relative abundance of bacteria at genus level (E) in individual mice and (F) combined mice by genotype. *n *= 9 each genotype. **Fig. S3.** Co-housed Phb1ΔPCmice manifest PC abnormalities and ileitis. (A) H&E staining showing Paneth cells (pink granules) in the ileal crypts. Bar = 50 μm; boxed pullout bar = 200 μm. (B) Immunofluorescent-staining for lysozyme (red), muc2 (green), and DAPI (nucleus, blue) in ileal crypts (dashed line). Arrows denote Paneth cells with normal lysozyme packaging into granules. Bar = 50 μm. (C) Average number of lysozyme+cells per crypt per mouse. A minimum of 50 crypts per mouse were quantitated. *n *= 13 Phb1ΔPCor 10 Phb1fl/fllittermates. (D) Paneth cell lysozyme allocation patterns. A minimum of 50 crypts per mouse were quantitated. *n *= 13 Phb1ΔPCor 10 Phb1fl/fllittermates. (E) mRNA quantification in ileum by qRT-PCR. *n*= 14 each genotype. (F) AB-PAS staining of ileum and number of AB-PAS+cells/crypt base. Bar = 100 μm. Dashed line denotes crypt base. *n *= 13 Phb1ΔPCor 10 Phb1fl/fllittermates. (G) Representative H&E-stained ileum and histological inflammation scoring of ileum. Bar = 100 μm. *n*= 21 Phb1ΔPCor 10 Phb1fl/fllittermates. (H) mRNA quantification in ileum by qRT-PCR. *n *= 14 each genotype. AB-PAS, alcian blue-periodic acid Schiff; Ang4, angiogenin 4; H&E, hematoxylin & eosin; Ifng, interferon gamma; Il1b, interleukin 1 beta; muc2, mucin2; Tnfa, tumor necrosis factor alpha. Results are presented as individual mice ±SD. *P< 0.05, **P< 0.01, ***P< 0.001, ****P< 0.0001. ****P< 0.0001. **Fig. S4.** Phb1iΔIECmice co-housed with Phb1fl/fllittermates exhibit Paneth cell defects and spontaneous ileitis. (A) H&E staining showing Paneth cells (pink granules) in the ileal crypts. Bar = 50 μm. (B) Immunofluorescent-staining for lysozyme (red), muc2 (green), and DAPI (nucleus, blue) in ileal crypts (dashed line). Arrows denote Paneth cells with normal lysozyme packaging into granules. Bar = 50 μm. (C) Average number of lysozyme+cells per crypt per mouse. *n *= 14 Phb1fl/fland 18 Phb1ΔPCmice. (D) Paneth cell lysozyme allocation patterns. *n *= 16 each genotype. (E)mRNA quantification in ileum by qRT-PCR. *n *=15 Phb1fl/fland 14 Phb1ΔPCmice.Outliers were identified by ROUT test (Q = 1%) and were removed fromPhb1fl/flAng4and Phb1ΔPCCryptdin3, Cryptdin 5. (F) AB-PAS staining of ileum and number of AB-PAS+cells/crypt base. Bar = 100 μm. Dashed line denotes crypt base. *n*= 15 each genotype. (G) Histological inflammation scoring of ileum. Arrow denotes infiltrating immune cells. Bar = 100 μm. *n *= 15 each genotype. AB-PAS, calcian blue-periodic acid Schiff; Ang4, angiogenin 4; H&E, hematoxylin & eosin; muc2, mucin2. Results are presented as individual mice ±SD. **P< 0.01, ***P< 0.001, ****P< 0.0001. **Fig. S5.** Butyrate supplementation prevents Paneth cell defects in Phb1-deficient mice. (A-B) Immunofluorescent-staining for lysozyme (red), muc2 (green), and DAPI (nucleus, blue) in ileal crypts (white outline) of Phb1ΔPCmice and Phb1fl/fllittermates (A) or Phb1iΔIECmice and Phb1fl/fllittermates (B). Arrows denote Paneth cells with normal Lysozyme packaging into granules. Asterisk denotes Muc2 colocalization with Lysozyme. Bar = 50 μm. (C-D)AB-PAS staining of ileum. Bar = 100 μm. Dashed line denotes crypt base. AB-PAS, alcian blue-periodic acid Schiff; muc2, mucin2; Veh, vehicle. **Fig. S6.** Butyrate protects Phb1-deficient ileal enteroids from Phb1iΔIEC microbiota-induced death. (A) Western blots for total and cleaved Caspase 3 indicating apoptosis. (B) Oxygen consumption relative to Phb1fl/flvehicle enteroids. As a positive control, enteroids were treated with 0.1 μM Antimycin A 15 min prior to oxygen measurements. *n *= 3 for all treatments. (C) Mitochondrial superoxide level as measured by MitoSOX fluorescence intensity. *n* = 3 for all treatments, results are representative of 2 separate experiments. C: Phb1fl/fl microbiota filtrate, KO: Phb1iΔIEC microbiota filtrate. *n *= 3 for all treatments. AntiA, Antimycin A. Results are presented as individual mice ± SD. *P< 0.05, **P< 0.01, ***P< 0.005, ****P< 0.0001.

## Data Availability

The 16S rRNA gene sequencing raw sequence reads (fastq) have been deposited in the NCBI Sequence Read Archive under project numbers PRJNA950478, PRJNA950917, and PRJNA951000.

## References

[1] Torres J, Mehandru S, Colombel JF, Peyrin-Biroulet L (2017). Crohn’s disease. Lancet.

[2] Ananthakrishnan AN, Kaplan GG, Ng SC (2020). Changing global epidemiology of inflammatory bowel diseases: sustaining health care delivery into the 21st century. Clin Gastroenterol Hepatol.

[3] Qiu P, Ishimoto T, Fu L, Zhang J, Zhang Z, Liu Y (2022). The gut microbiota in inflammatory bowel disease. Front Cell Infect Microbiol.

[4] Shan Y, Lee M, Chang EB (2022). The gut microbiome and inflammatory bowel diseases. Annu Rev Med.

[5] Wallaeys C, Garcia-Gonzalez N, Libert C (2023). Paneth cells as the cornerstones of intestinal and organismal health: a primer. EMBO Mol Med.

[6] Wehkamp J, Schmid M, Fellermann K, Stange EF (2005). Defensin deficiency, intestinal microbes, and the clinical phenotypes of Crohn’s disease. J Leukoc Biol.

[7] Yang E, Shen J (2021). The roles and functions of Paneth cells in Crohn’s disease: a critical review. Cell Prolif.

[8] Parada Venegas D, De la Fuente MK, Landskron G, Gonzalez MJ, Quera R, Dijkstra G, Harmsen HJM (2019). Short chain fatty acids (SCFAs)-mediated gut epithelial and immune regulation and its relevance for inflammatory bowel diseases. Front Immunol.

[9] Park JH, Kotani T, Konno T, Setiawan J, Kitamura Y, Imada S, Usui Y (2016). Promotion of intestinal epithelial cell turnover by commensal bacteria: role of short-chain fatty acids. PLoS ONE.

[10] Salvi PS, Cowles RA (2021). Butyrate and the intestinal epithelium: modulation of proliferation and inflammation in homeostasis and disease. Cells.

[11] Laserna-Mendieta EJ, Clooney AG, Carretero-Gomez JF, Moran C, Sheehan D, Nolan JA, Hill C (2018). Determinants of reduced genetic capacity for butyrate synthesis by the gut microbiome in Crohn’s disease and ulcerative colitis. J Crohns Colitis.

[12] Haberman Y, Karns R, Dexheimer PJ, Schirmer M, Somekh J, Jurickova I, Braun T (2019). Ulcerative colitis mucosal transcriptomes reveal mitochondriopathy and personalized mechanisms underlying disease severity and treatment response. Nat Commun.

[13] Ho GT, Aird RE, Liu B, Boyapati RK, Kennedy NA, Dorward DA, Noble CL (2018). MDR1 deficiency impairs mitochondrial homeostasis and promotes intestinal inflammation. Mucosal Immunol.

[14] Kugathasan S, Denson LA, Walters TD, Kim MO, Marigorta UM, Schirmer M, Mondal K (2017). Prediction of complicated disease course for children newly diagnosed with Crohn’s disease: a multicentre inception cohort study. Lancet.

[15] Mottawea W, Chiang CK, Muhlbauer M, Starr AE, Butcher J, Abujamel T, Deeke SA (2016). Altered intestinal microbiota-host mitochondria crosstalk in new onset Crohn’s disease. Nat Commun.

[16] Rath E, Berger E, Messlik A, Nunes T, Liu B, Kim SC, Hoogenraad N (2012). Induction of dsRNA-activated protein kinase links mitochondrial unfolded protein response to the pathogenesis of intestinal inflammation. Gut.

[17] Colgan SP, Wang RX, Hall CHT, Bhagavatula G, Lee JS (2023). Revisiting the “starved gut” hypothesis in inflammatory bowel disease. Immunometabolism (Cobham).

[18] Roediger WE (1980). The colonic epithelium in ulcerative colitis: an energy-deficiency disease?. Lancet.

[19] Alula KM, Jackson DN, Smith AD, Kim DS, Turner K, Odstrcil E (2021). Targeting mitochondrial damage as a therapeutic for ileal Crohn’s disease. Cells.

[20] Jackson DN, Panopoulos M, Neumann WL, Turner K, Cantarel BL, Thompson-Snipes L, Dassopoulos T (2020). Mitochondrial dysfunction during loss of prohibitin 1 triggers Paneth cell defects and ileitis. Gut.

[21] Hsieh SY, Shih TC, Yeh CY, Lin CJ, Chou YY, Lee YS (2006). Comparative proteomic studies on the pathogenesis of human ulcerative colitis. Proteomics.

[22] Nijtmans LG, de Jong L, Artal Sanz M, Coates PJ, Berden JA, Back JW, Muijsers AO (2000). Prohibitins act as a membrane-bound chaperone for the stabilization of mitochondrial proteins. EMBO J.

[23] Theiss AL, Idell RD, Srinivasan S, Klapproth JM, Jones DP, Merlin D, Sitaraman SV (2007). Prohibitin protects against oxidative stress in intestinal epithelial cells. FASEB J.

[24] Liu TC, Gao F, McGovern DP, Stappenbeck TS (2014). Spatial and temporal stability of paneth cell phenotypes in Crohn’s disease: implications for prognostic cellular biomarker development. Inflamm Bowel Dis.

[25] Liu TC, Gurram B, Baldridge MT, Head R, Lam V, Luo C, Cao Y (2016). Paneth cell defects in Crohn’s disease patients promote dysbiosis. JCI Insight.

[26] VanDussen KL, Liu TC, Li D, Towfic F, Modiano N, Winter R, Haritunians T (2014). Genetic variants synthesize to produce paneth cell phenotypes that define subtypes of Crohn’s disease. Gastroenterology.

[27] el Marjou F, Janssen KP, Chang BH, Li M, Hindie V, Chan L, Louvard D (2004). Tissue-specific and inducible Cre-mediated recombination in the gut epithelium. Genesis.

[28] Lotti C, Rubert J, Fava F, Tuohy K, Mattivi F, Vrhovsek U (2017). Development of a fast and cost-effective gas chromatography-mass spectrometry method for the quantification of short-chain and medium-chain fatty acids in human biofluids. Anal Bioanal Chem.

[29] Wang RX, Lee JS, Campbell EL, Colgan SP (2020). Microbiota-derived butyrate dynamically regulates intestinal homeostasis through regulation of actin-associated protein synaptopodin. Proc Natl Acad Sci U S A.

[30] Sato T, Vries RG, Snippert HJ, van de Wetering M, Barker N, Stange DE, van Es JH (2009). Single Lgr5 stem cells build crypt-villus structures in vitro without a mesenchymal niche. Nature.

[31] Qian BF, Tonkonogy SL, Hoentjen F, Dieleman LA, Sartor RB (2005). Dysregulated luminal bacterial antigen-specific T-cell responses and antigen-presenting cell function in HLA-B27 transgenic rats with chronic colitis. Immunology.

[32] Grabinger T, Luks L, Kostadinova F, Zimberlin C, Medema JP, Leist M, Brunner T (2014). Ex vivo culture of intestinal crypt organoids as a model system for assessing cell death induction in intestinal epithelial cells and enteropathy. Cell Death Dis.

[33] Kelly CJ, Zheng L, Campbell EL, Saeedi B, Scholz CC, Bayless AJ, Wilson KE (2015). Crosstalk between microbiota-derived short-chain fatty acids and intestinal epithelial HIF augments tissue barrier function. Cell Host Microbe.

[34] Burns RC, Rivera-Nieves J, Moskaluk CA, Matsumoto S, Cominelli F, Ley K (2001). Antibody blockade of ICAM-1 and VCAM-1 ameliorates inflammation in the SAMP-1/Yit adoptive transfer model of Crohn’s disease in mice. Gastroenterology.

[35] Arpaia N, Campbell C, Fan X, Dikiy S, van der Veeken J, deRoos P, Liu H (2013). Metabolites produced by commensal bacteria promote peripheral regulatory T-cell generation. Nature.

[36] Maslowski KM, Vieira AT, Ng A, Kranich J, Sierro F, Yu D (2009). Regulation of inflammatory responses by gut microbiota and chemoattractant receptor GPR43. Nature.

[37] Smith PM, Howitt MR, Panikov N, Michaud M, Gallini CA, Bohlooly YM, Glickman JN (2013). The microbial metabolites, short-chain fatty acids, regulate colonic Treg cell homeostasis. Science.

[38] Ruppin H, Bar-Meir S, Soergel KH, Wood CM, Schmitt MG (1980). Absorption of short-chain fatty acids by the colon. Gastroenterology.

[39] Lukovac S, Belzer C, Pellis L, Keijser BJ, de Vos WM, Montijn RC, Roeselers G. Differential modulation by Akkermansia muciniphila and Faecalibacterium prausnitzii of host peripheral lipid metabolism and histone acetylation in mouse gut organoids. mBio. 2014;5. 10.1128/mBio.01438-14 PMC4145684 25118238

[40] Turrens JF (2003). Mitochondrial formation of reactive oxygen species. J Physiol.

[41] Gasaly N, Hermoso MA, Gotteland M. Butyrate and the fine-tuning of colonic homeostasis: implication for inflammatory bowel diseases. Int J Mol Sci. 2021;22. 10.3390/ijms22063061 PMC8002420 33802759

[42] Davie JR (2003). Inhibition of histone deacetylase activity by butyrate. J Nutr.

[43] Walker GE, Wilson EM, Powell D, Oh Y (2001). Butyrate, a histone deacetylase inhibitor, activates the human IGF binding protein-3 promoter in breast cancer cells: molecular mechanism involves an Sp1/Sp3 multiprotein complex. Endocrinology.

[44] Finegold SM (1969). Intestinal bacteria. The role they play in normal physiology, pathologic physiology, and infection. Calif Med.

[45] Donaldson GP, Lee SM, Mazmanian SK (2016). Gut biogeography of the bacterial microbiota. Nat Rev Microbiol.

[46] Gevers D, Kugathasan S, Denson LA, Vazquez-Baeza Y, Van Treuren W, Ren B, Schwager E (2014). The treatment-naive microbiome in new-onset Crohn’s disease. Cell Host Microbe.

[47] Lopez-Siles M, Martinez-Medina M, Busquets D, Sabat-Mir M, Duncan SH, Flint HJ, Aldeguer X (2014). Mucosa-associated Faecalibacterium prausnitzii and Escherichia coli co-abundance can distinguish irritable bowel syndrome and inflammatory bowel disease phenotypes. Int J Med Microbiol.

[48] Jackson DN, Theiss AL (2020). Gut bacteria signaling to mitochondria in intestinal inflammation and cancer. Gut Microbes.

[49] Schaubeck M, Clavel T, Calasan J, Lagkouvardos I, Haange SB, Jehmlich N, Basic M (2016). Dysbiotic gut microbiota causes transmissible Crohn’s disease-like ileitis independent of failure in antimicrobial defence. Gut.

[50] Amorim N, McGovern E, Raposo A, Khatiwada S, Shen S, Koentgen S, Hold G (2022). Refining a protocol for faecal microbiota engraftment in animal models after successful antibiotic-induced gut decontamination. Front Med (Lausanne).

[51] Bokoliya SC, Dorsett Y, Panier H, Zhou Y (2021). Procedures for fecal microbiota transplantation in murine microbiome studies. Front Cell Infect Microbiol.

[52] Di Sabatino A, Morera R, Ciccocioppo R, Cazzola P, Gotti S, Tinozzi FP (2005). Oral butyrate for mildly to moderately active Crohn’s disease. Aliment Pharmacol Ther.

[53] Huang X, Oshima T, Tomita T, Fukui H, Miwa H. Butyrate alleviates cytokine-induced barrier dysfunction by modifying claudin-2 levels. Biology (Basel). 2021;10. 10.3390/biology10030205 PMC8000923 33803334

[54] Pena-Rodriguez M, Vega-Magana N, Garcia-Benavides L, Zepeda-Nuno JS, Gutierrez-Silerio GY, Gonzalez-Hernandez LA, Andrade-Villanueva JF (2022). Butyrate administration strengthens the intestinal epithelium and improves intestinal dysbiosis in a cholestasis fibrosis model. J Appl Microbiol.

[55] Takakuwa A, Nakamura K, Kikuchi M, Sugimoto R, Ohira S, Yokoi Y, Ayabe T. Butyric acid and leucine induce alpha-defensin secretion from small intestinal Paneth cells. Nutrients. 2019;11. 10.3390/nu11112817 PMC6893607 31752111

