# Supplementary material for: Interplay of gut microbiota and host epithelial mitochondrial dysfunction is necessary for the development of spontaneous intestinal inflammation in mice
Source: Microbiome. 2023 Nov 17;11:256. doi: 10.1186/s40168-023-01686-9 (PMC10655390; doi:10.1186/s40168-023-01686-9)
Supplement: Supplementary file 2 — Additional file 1: Fig. S1. Elimination of gut microbiota by broad-spectrum antibiotics (ABX) prevents the manifestation of crypt base AB-PAS+cells in Phb1 deficient mice. (A) Bacterial elimination by ABX shown by reads per sample by 16S rRNA sequencing. (B) AB-PAS staining of ileum. Bar = 100 μm. Dashed line denotes crypt base. (C) Number of AB-PAS+cells/crypt base across 50 crypts.AB-PAS, alcian blue-periodic acid Schiff. n = 7-13 each group. ****P< 0.0001. Fig. S2. Co-housed Phb1ΔPC mice exhibit similar ileal microbiota composition as Phb1fl/fl littermates.16S RNA sequencing of luminal ileal content of co-housed Phb1fl/fl and Phb1ΔPC mice. (A) Alpha diversity measure by Shannon index. (B) Weighted UniFrac PCoA plot of 16S rRNA gene sequences. (C-D) Relative abundance of bacteria at phylum level (C) in individual mice and (D) combined mice by genotype. (E-F) Relative abundance of bacteria at genus level (E) in individual mice and (F) combined mice by genotype. n = 9 each genotype. Fig. S3. Co-housed Phb1ΔPCmice manifest PC abnormalities and ileitis. (A) H&E staining showing Paneth cells (pink granules) in the ileal crypts. Bar = 50 μm; boxed pullout bar = 200 μm. (B) Immunofluorescent-staining for lysozyme (red), muc2 (green), and DAPI (nucleus, blue) in ileal crypts (dashed line). Arrows denote Paneth cells with normal lysozyme packaging into granules. Bar = 50 μm. (C) Average number of lysozyme+cells per crypt per mouse. A minimum of 50 crypts per mouse were quantitated. n = 13 Phb1ΔPCor 10 Phb1fl/fllittermates. (D) Paneth cell lysozyme allocation patterns. A minimum of 50 crypts per mouse were quantitated. n = 13 Phb1ΔPCor 10 Phb1fl/fllittermates. (E) mRNA quantification in ileum by qRT-PCR. n= 14 each genotype. (F) AB-PAS staining of ileum and number of AB-PAS+cells/crypt base. Bar = 100 μm. Dashed line denotes crypt base. n = 13 Phb1ΔPCor 10 Phb1fl/fllittermates. (G) Representative H&E-stained ileum and histological inflammation scoring of ileum. Bar = 100 μm [file 40168_2023_1686_MOESM1_ESM.pdf]

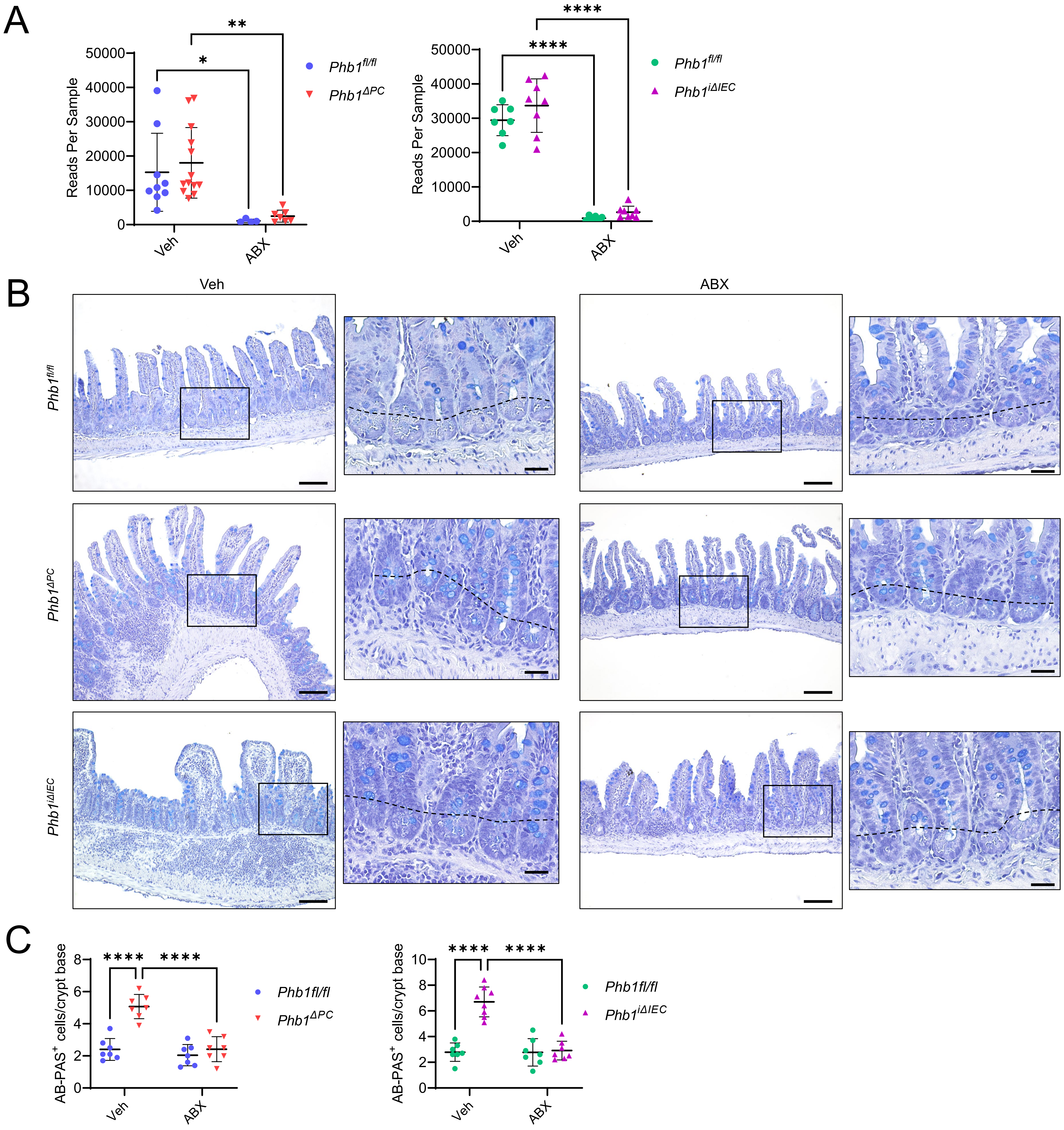

Supplemental Figure S1. Elimination of gut microbiota by broad-spectrum antibiotics (ABX) prevents the manifestation of crypt base AB-PAS⁺ cells in *Phb1* deficient mice. (A) Bacterial elimination by ABX shown by reads per sample by 16S rRNA sequencing. (B) AB-PAS staining of ileum. Bar = 100 µm. Dashed line denotes crypt base. (C) Number of AB-PAS⁺ cells/crypt base across 50 crypts. AB-PAS, alcian blue-periodic acid Schiff. n = 7-13 each group. ****P < 0.0001.

Co-housed across genotype

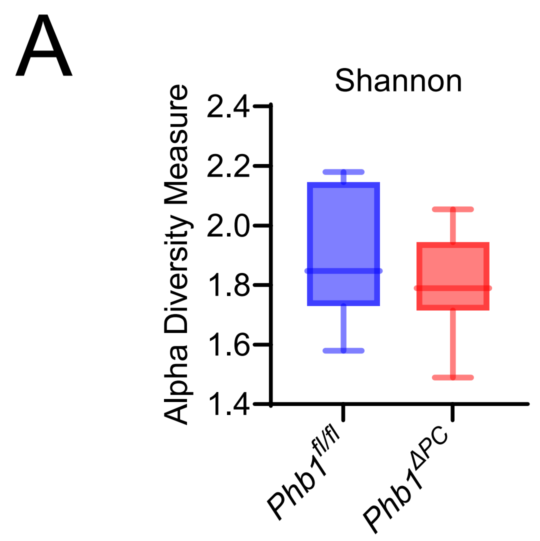

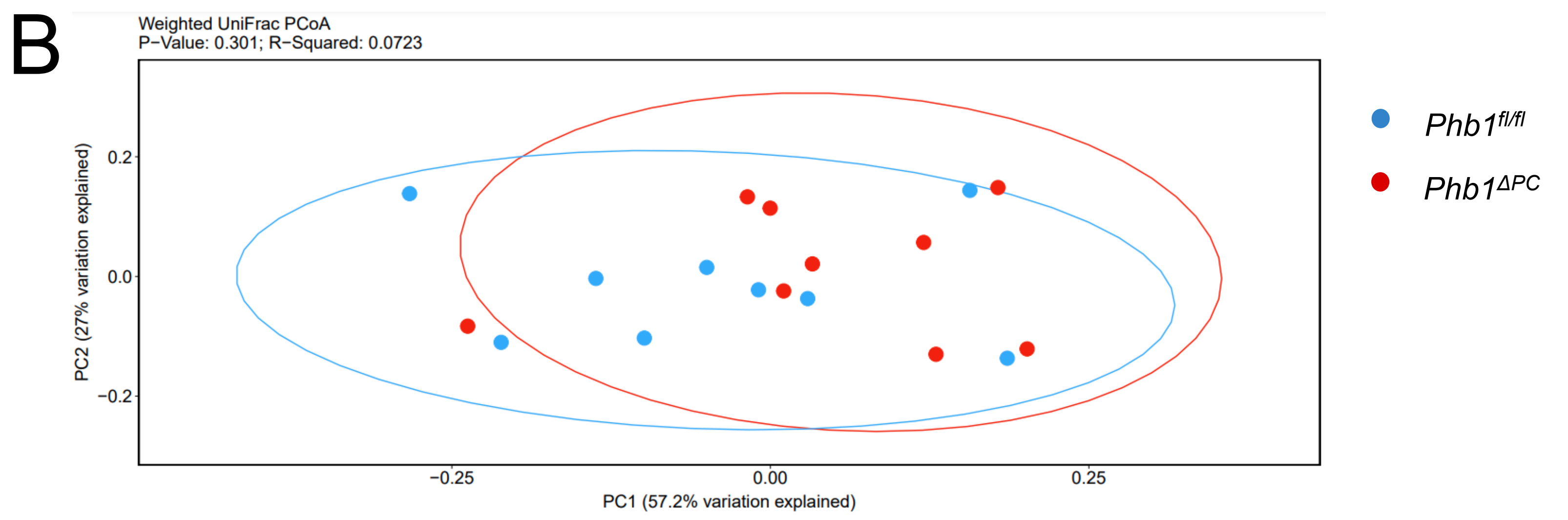

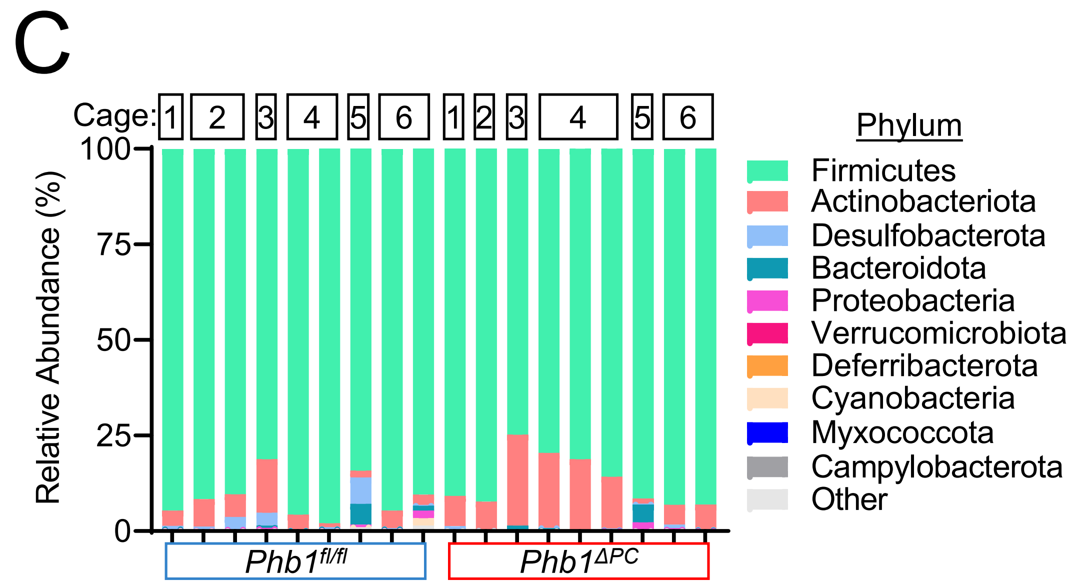

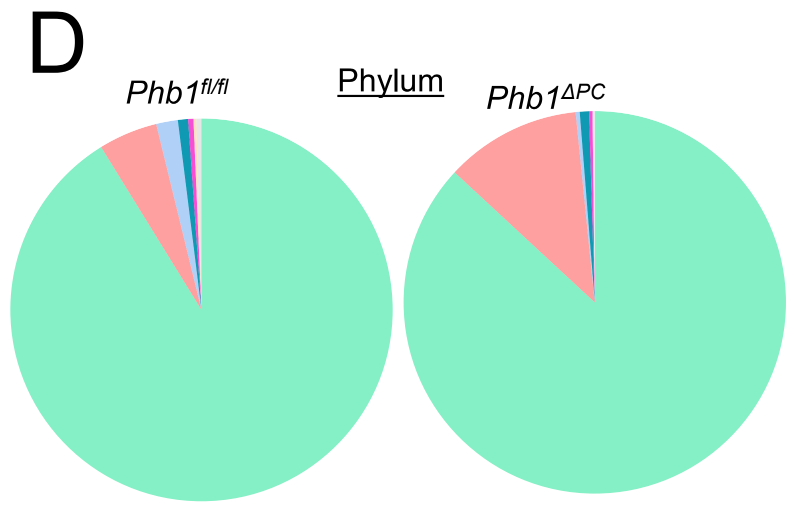

Phylum (%)	*Phb1 fl/fl*	*Phb1 ΔPC*
Firmicutes	91.2	86.9
Actinobacteriota	5	11.5
Desulfobacterota	1.8	0.3
Bacteroidota	0.9	0.8
Proteobacteria	0.5	0.3
Verrucomicrobiota	0	0
Defferibacterota	0	0
Cyanobacteria	0.2	0
Myxococcota	0	0
Campylobacterota	0	0
Other	0.4	0.2

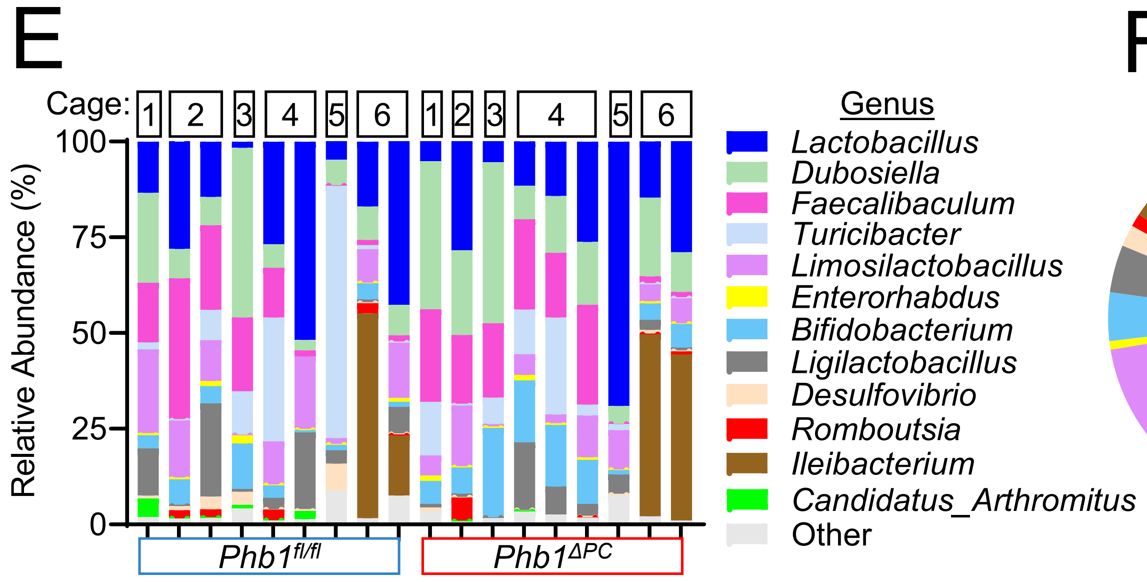

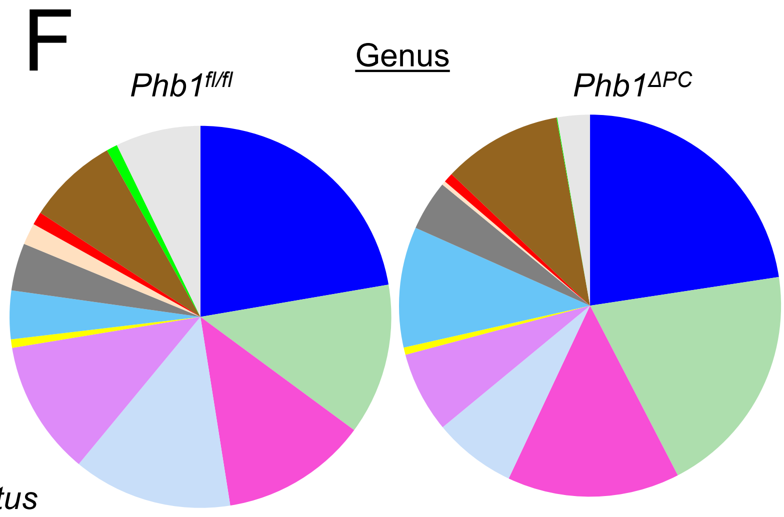

Genus (%)	*Phb1 fl/fl*	*Phb1 ΔPC*
Lactobacillus	22.3	22.6
Dubosiella	12.8	19.8
Faecalibaculum	12.4	14.6
Turicibacter	13.5	7.0
Limosilactobacillus	11.4	6.8
Enterorhabdus	0.7	0.6
Bifidobacterium	4.1	10.2
Ligilactobacillus	4.0	4.3
Desulfovibrio	1.8	0.3
Romboutsia	1.1	0.8
Ileibacterium	7.7	10.1
Candidatus_Arthromitus	0.9	0.06
Other	7.2	2.8

Supplemental Figure S2. Co-housed *Phb1$^{\Delta PC}$* mice exhibit similar ileal microbiota composition as *Phb1$^{fl/fl}$* littermates. 16S RNA sequencing of luminal ileal content of co-housed *Phb1$^{fl/fl}$* and *Phb1$^{\Delta PC}$* mice. (A) Alpha diversity measure by Shannon index. (B) Weighted UniFrac PCoA plot of 16S rRNA gene sequences. (C-D) Relative abundance of bacteria at phylum level (C) in individual mice and (D) combined mice by genotype. (E-F) Relative abundance of bacteria at genus level (E) in individual mice and (F) combined mice by genotype. *n* = 9 each genotype.

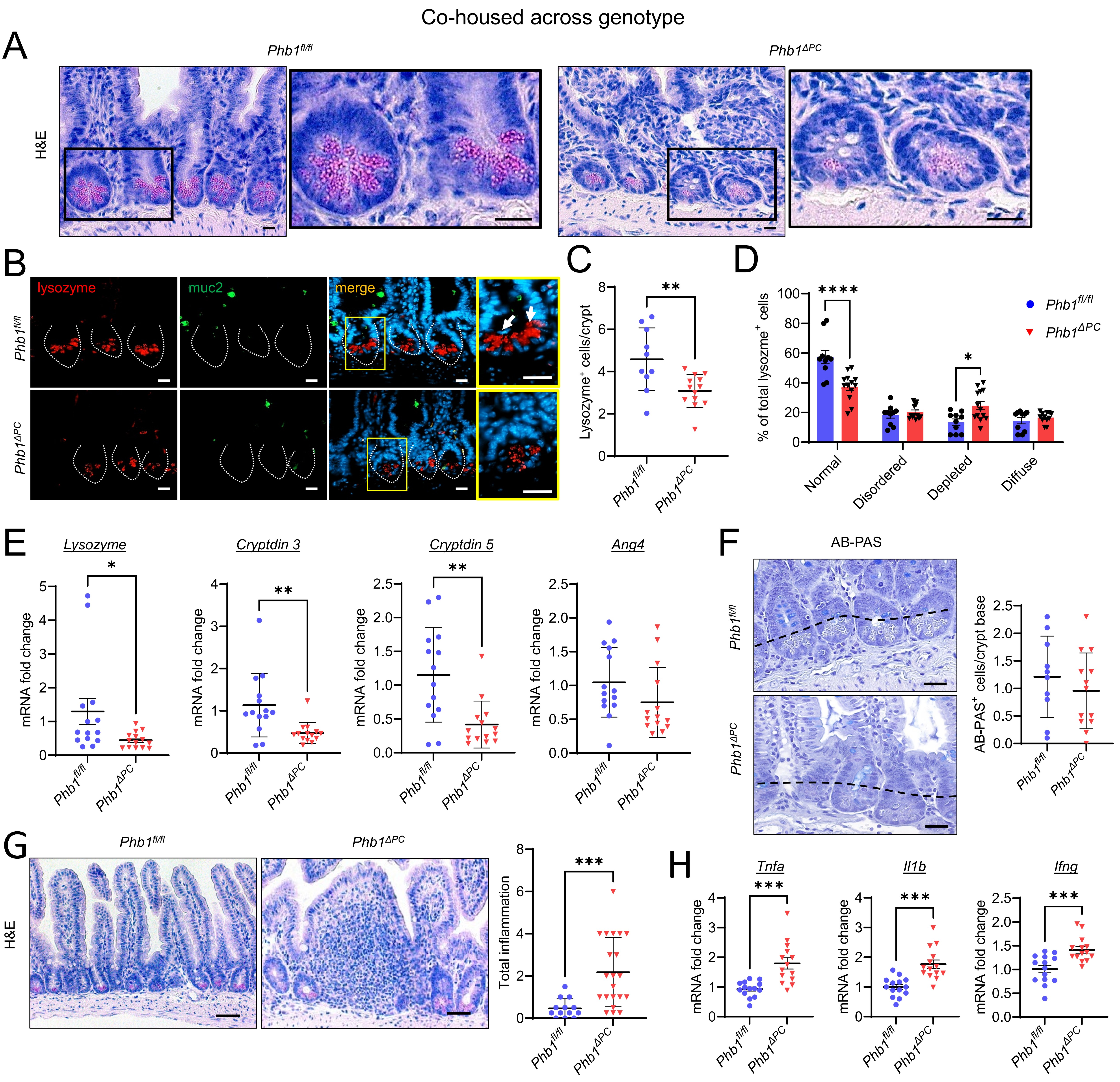

Supplemental Figure S3. Co-housed *Phb1*$^{\Delta PC}$ mice manifest PC abnormalities and ileitis. (A) H&E staining showing Paneth cells (pink granules) in the ileal crypts. Bar = 50 µm; boxed pullout bar = 200 µm. (B) Immunofluorescent-staining for lysozyme (red), muc2 (green), and DAPI (nucleus, blue) in ileal crypts (dashed line). Arrows denote Paneth cells with normal lysozyme packaging into granules. Bar = 50 µm. (C) Average number of lysozyme$^+$ cells per crypt per mouse. A minimum of 50 crypts per mouse were quantitated. *n* = 13 *Phb1*$^{\Delta PC}$ or 10 *Phb1*$^{fl/fl}$ littermates. (D) Paneth cell lysozyme allocation patterns. A minimum of 50 crypts per mouse were quantitated. *n* = 13 *Phb1*$^{\Delta PC}$ or 10 *Phb1*$^{fl/fl}$ littermates. (E) mRNA quantification in ileum by qRT-PCR. *n* = 14 each genotype. (F) AB-PAS staining of ileum and number of AB-PAS$^+$ cells/crypt base. Bar = 100 µm. Dashed line denotes crypt base. *n* = 13 *Phb1*$^{\Delta PC}$ or 10 *Phb1*$^{fl/fl}$ littermates. (G) Representative H&E-stained ileum and histological inflammation scoring of ileum. Bar = 100 µm. *n* = 21 *Phb1*$^{\Delta PC}$ or 10 *Phb1*$^{fl/fl}$ littermates. (H) mRNA quantification in ileum by qRT-PCR. *n* = 14 each genotype. AB-PAS, alcian blue-periodic acid Schiff; Ang4, angiogenin 4; H&E, hematoxylin & eosin; Ifng, interferon gamma; Il1b, interleukin 1 beta; muc2, mucin2; Tnfa, tumor necrosis factor alpha. Results are presented as individual mice ± SD. *P < 0.05, **P < 0.01, ***P < 0.001, ****P < 0.0001.. ****P < 0.0001.

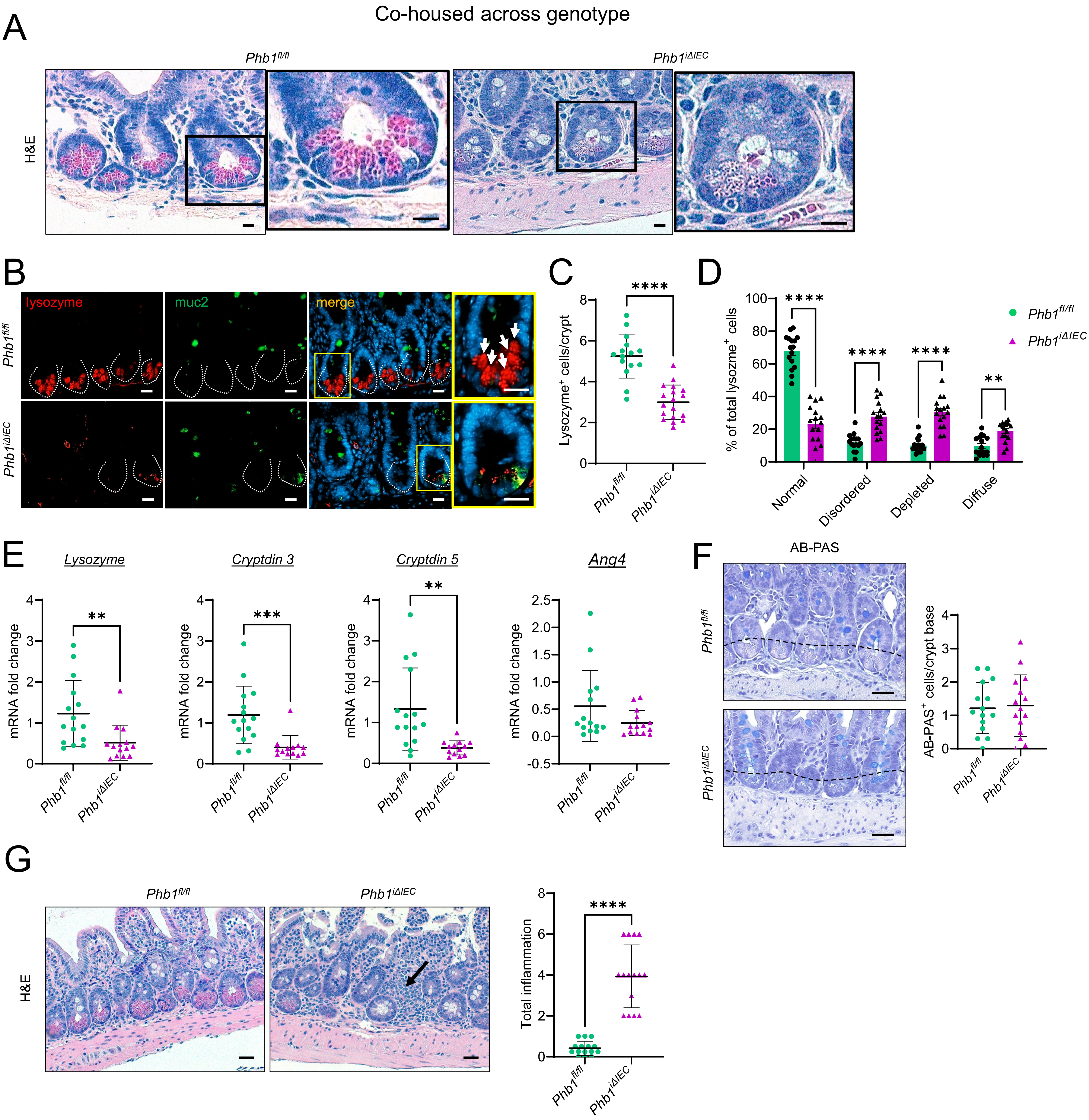

Supplemental Figure S4. $Phb1^{i\Delta IEC}$ mice co-housed with $Phb1^{fl/fl}$ littermates exhibit Paneth cell defects and spontaneous ileitis. (A) H&E staining showing Paneth cells (pink granules) in the ileal crypts. Bar = 50 µm. (B) Immunofluorescent-staining for lysozyme (red), muc2 (green), and DAPI (nucleus, blue) in ileal crypts (dashed line). Arrows denote Paneth cells with normal lysozyme packaging into granules. Bar = 50 µm. (C) Average number of lysozyme$^+$ cells per crypt per mouse. *n* = 14 $Phb1^{fl/fl}$ and 18 $Phb1^{\Delta PC}$ mice. (D) Paneth cell lysozyme allocation patterns. *n* = 16 each genotype. (E) mRNA quantification in ileum by qRT-PCR. *n* = 15 $Phb1^{fl/fl}$ and 14 $Phb1^{\Delta PC}$ mice. Outliers were identified by ROUT test (Q = 1%) and were removed from $Phb1^{fl/fl}$ *Ang4* and $Phb1^{\Delta PC}$ *Cryptdin3*, *Cryptdin 5*. (F) AB-PAS staining of ileum and number of AB-PAS$^+$ cells/crypt base. Bar = 100 µm. Dashed line denotes crypt base. *n* = 15 each genotype. (G) Histological inflammation scoring of ileum. Arrow denotes infiltrating immune cells. Bar = 100 µm. *n* = 15 each genotype. AB-PAS, calcian blue-periodic acid Schiff; Ang4, angiogenin 4; H&E, hematoxylin & eosin; muc2, mucin2. Results are presented as individual mice ± SD. **$P < 0.01$, ***$P < 0.001$, ****$P < 0.0001$.

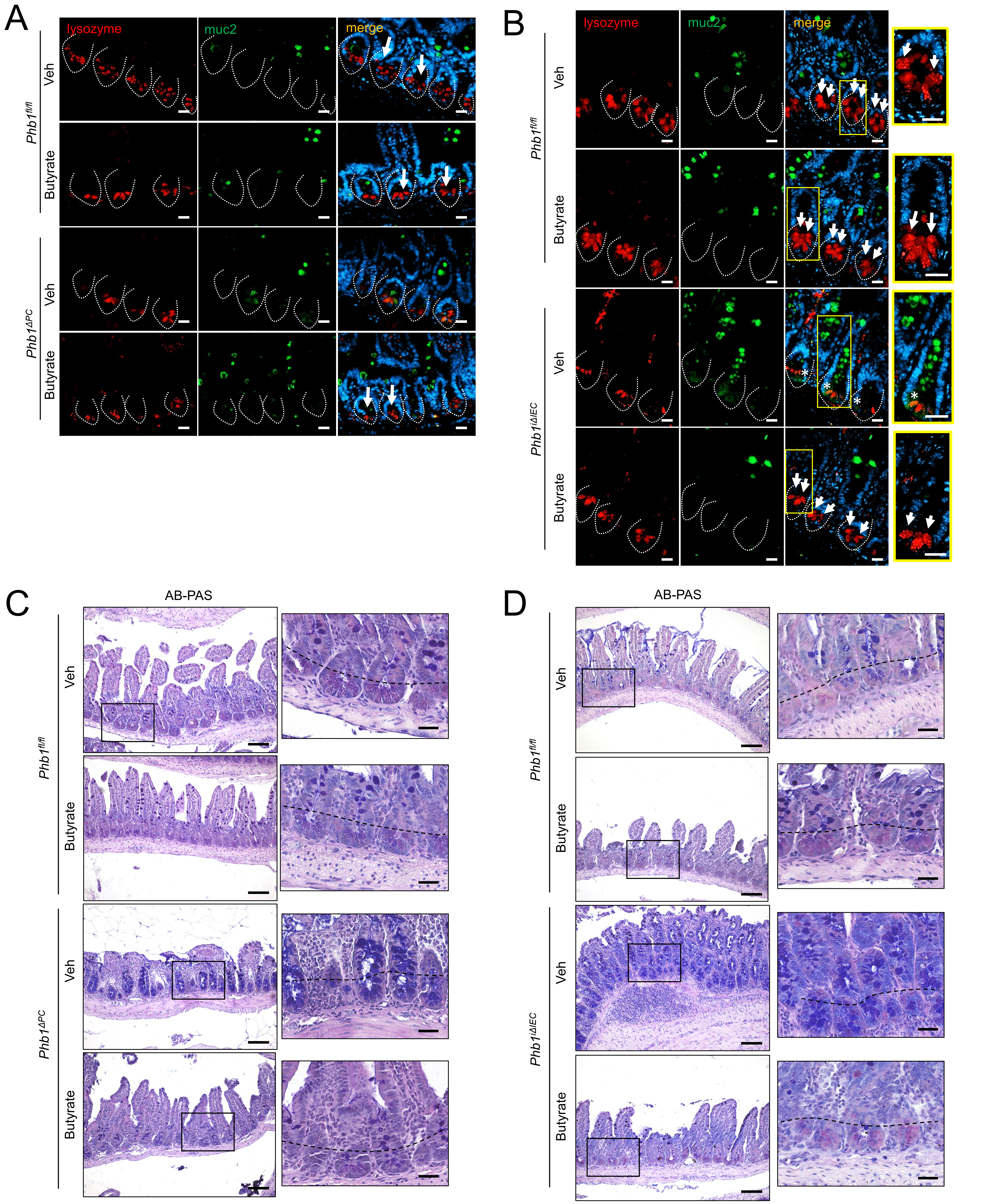

Supplemental Figure S5. Butyrate supplementation prevents Paneth cell defects in *Phb1*-deficient mice. (A-B) Immunofluorescent-staining for lysozyme (red), muc2 (green), and DAPI (nucleus, blue) in ileal crypts (white outline) of $Phb1^{\Delta PC}$ mice and $Phb1^{fl/fl}$ littermates (A) or $Phb1^{i\Delta IEC}$ mice and $Phb1^{fl/fl}$ littermates (B). Arrows denote Paneth cells with normal Lysozyme packaging into granules. Asterisk denotes Muc2 colocalization with Lysozyme. Bar = 50 μm. (C-D) AB-PAS staining of ileum. Bar = 100 μm. Dashed line denotes crypt base. AB-PAS, alcian blue-periodic acid Schiff; muc2, mucin2; Veh, vehicle

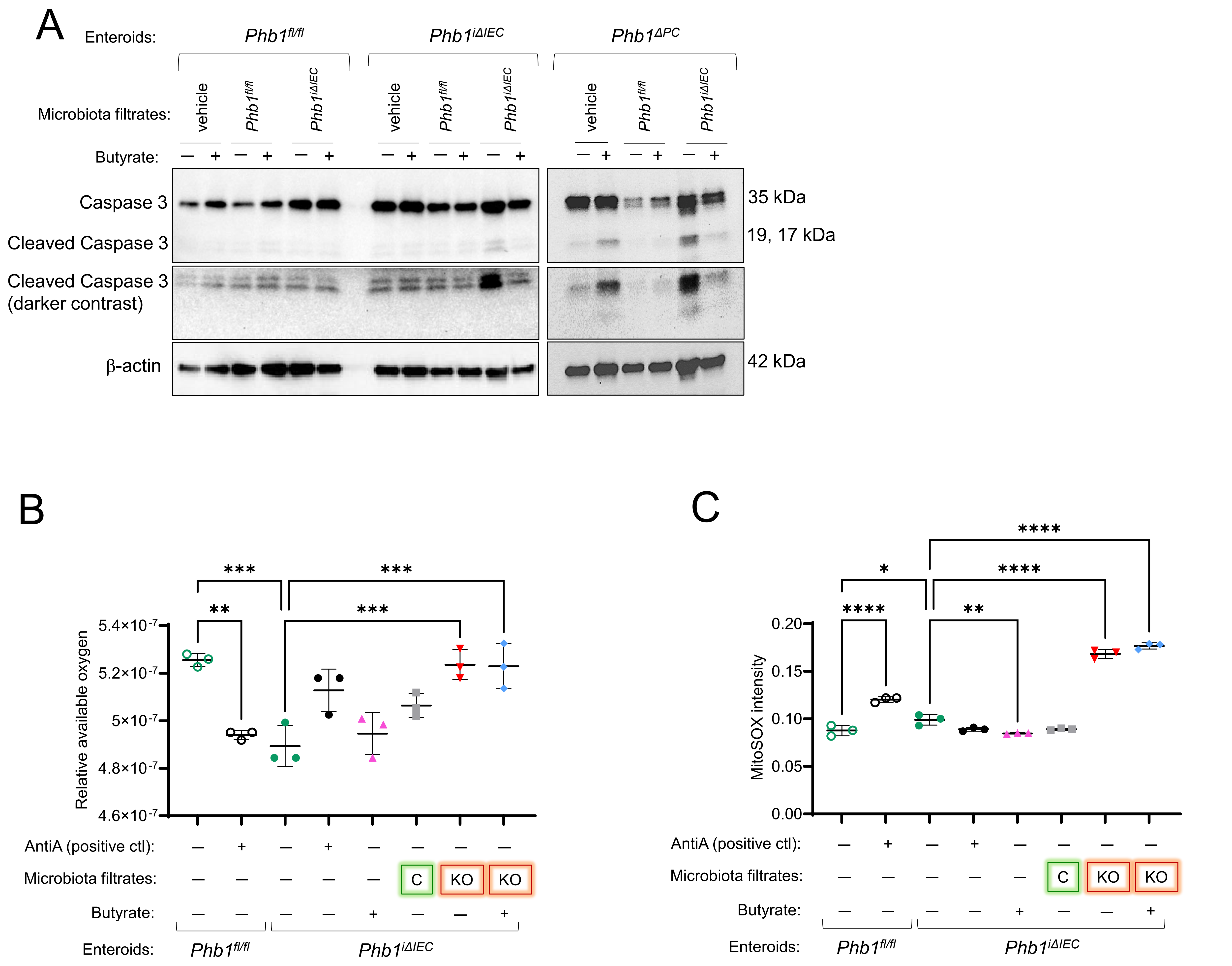

Supplemental Figure S6. Butyrate protects *Phb1*-deficient ileal enteroids from $Phb1^{i\Delta IEC}$ microbiota-induced death. (A) Western blots for total and cleaved Caspase 3 indicating apoptosis. (B) Oxygen consumption relative to $Phb1^{fl/fl}$ vehicle enteroids. As a positive control, enteroids were treated with 0.1 μM Antimycin A 15 min prior to oxygen measurements. n = 3 for all treatments. (C) Mitochondrial superoxide level as measured by MitoSOX fluorescence intensity. n = 3 for all treatments, results are representative of 2 separate experiments. C: $Phb1^{fl/fl}$ microbiota filtrate, KO: $Phb1^{i\Delta IEC}$ microbiota filtrate. n = 3 for all treatments. AntiA, Antimycin A. Results are presented as individual mice ± SD. *P < 0.05, **P < 0.01, ***P < 0.005, ****P < 0.0001.
